# Ratiometric Detection of Mercury (II) Ions in Living Cells Using Fluorescent Probe Based on Bis(styryl) Dye and Azadithia-15-Crown-5 Ether Receptor

**DOI:** 10.3390/s21020470

**Published:** 2021-01-11

**Authors:** Pavel A. Panchenko, Anastasija V. Efremenko, Alexey V. Feofanov, Mariya A. Ustimova, Yuri V. Fedorov, Olga A. Fedorova

**Affiliations:** 1 Laboratory of Photoactive Supramolecular systems, A.N. Nesmeyanov Institute of Organoelement Compounds of Russian Academy of Sciences (INEOS RAS), 119991 Moscow, Russia; ustimova.maria@yandex.ru (M.A.U.); fedorov@ineos.ac.ru (Y.V.F.); fedorova@ineos.ac.ru (O.A.F.); 2 Department of Technology of Fine Organic Synthesis and Chemistry of Dyes, Dmitry Mendeleev University of Chemical Technology of Russia, 125047 Moscow, Russia; 3 Biological Faculty, Lomonosov Moscow State University, 119992 Moscow, Russia; aefr@mail.ru (A.V.E.); avfeofanov@yandex.ru (A.V.F.); 4 Laboratory of Optical Microscopy and Spectroscopy, Shemyakin-Ovchinnikov Institute of Bioorganic Chemistry of Russian Academy of Sciences, 117997 Moscow, Russia

**Keywords:** Hg^2+^, ratiometric sensor, fluorescence imaging, resonance energy transfer, intramolecular charge transfer, styryl dye, crown ether, living cells, human lung adenocarcinoma A549 cells

## Abstract

Bis(styryl) dye **1** bearing *N*-phenylazadithia-15-crown-5 ether receptor has been evaluated as a ratiometric fluorescent chemosensor for mercury (II) ions in living cells. In aqueous solution, probe **1** selectively responds to the presence of Hg^2+^ via the changes in the emission intensity as well as in the emission band shape, which is a result of formation of the complex with 1:1 metal to ligand ratio (dissociation constant 0.56 ± 0.15 µM). The sensing mechanism is based on the interplay between the RET (resonance energy transfer) and ICT (intramolecular charge transfer) interactions occurring upon the UV/Vis (380 or 405 nm) photoexcitation of both styryl chromophores in probe 1. Bio-imaging studies revealed that the yellow (500–600 nm) to red (600–730 nm) fluorescence intensity ratio decreased from 4.4 ± 0.2 to 1.43 ± 0.10 when cells were exposed to increasing concentration of mercury (II) ions enabling ratiometric quantification of intracellular Hg^2+^ concentration in the 37 nM–1 μM range.

## 1. Introduction

Construction of fluorescent chemosensors for heavy and transition metal cations are in the focus of current research [1,2,3]. Among various cations, Hg^2+^ is well-known for its high toxicity and capacity for bioaccumulation. Hg^2+^ ions can be released in the environment along with the effluent or as a result of atmospheric oxidation of mercury vapor [4]. Further bioaccumulation by microorganisms in water transforms Hg^2+^ to methylmercury, a form that can be easily included into the food chain [5,6]. Upon consumption by humans, this toxin may cause serious health disorders [7]. Given that mercury and its compounds are extensively employed in industry and agriculture [8], reliable and sensitive probes for the detection of Hg^2+^ in biological systems are in demand.

To date, a large number of fluorescent chemosensors capable of tracing of low concentrations of Hg^2+^ in aqueous solutions has been reported. Most of them exhibit changes in the fluorescent characteristics due to switching of the photoinduced electron transfer (PET) process between the receptor and chromophore upon complexation with Hg^2+^. Such systems provide detection of analyte via enhancement [9,10] or quenching [11,12] of the emission intensity without changes in the shape and maximum position of a fluorescence spectrum. At the same time, intensity responsive sensors have limited applicability for Hg^2+^ detection in vivo, in particular, in living cells, because their intracellular fluorescence is affected by concentrations of both Hg^2+^ and sensor itself. Analytics in vivo requires ratiometric sensors, which change fluorescence spectrum shape and/or maximum upon complexation with Hg^2+^ [13]. Dual-wavelength measurement of fluorescence with ratiometric sensors obviates an influence of sensor concentration on the Hg^2+^ detection and facilitates Hg^2+^ quantification both in vivo and in vitro.

**Scheme 1:**
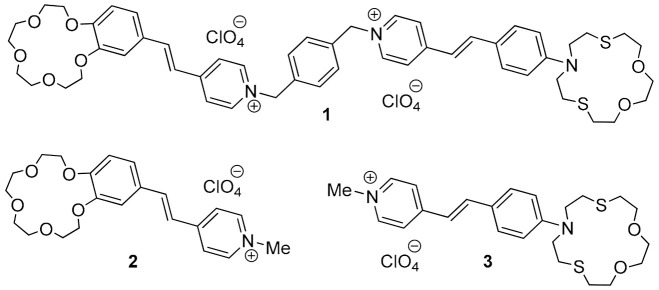
Chemical structure of compounds **1**, **2**, and **3**.

Ratiometric fluorescent response to metal cations can be realized by means of several photophysical mechanisms: intramolecular charge transfer (ICT) [14,15], resonance energy transfer (RET) between two chromophore fragments [16,17,18], excimer/exciplex formation [19], or excited state intramolecular proton transfer (ESIPT) [20]. Despite the obvious advantage, the number of ratiometric fluorescent probes for Hg^2+^ cation is rather limited even for in vitro analysis, and most of them exploit Hg^2+^-induced spyrolactam ring opening of rhodamine dyes, which act as energy acceptor chromophores [21,22,23]. Alternatively, we have recently demonstrated that coordination of metal ions (Na^+^, Li^+^, Mg^2+^, Ca^2+^, Ba^2+^, Hg^2+^, Ag^+^) and H^+^ with either azacrown or oxacrown receptors of bis(styryl) dye **1** (Scheme 1) in an organic solvent (acetonitrile) allows one to modulate the RET efficiency between the photoactive styryl subunits, thus producing a distinct ratiometric fluorescent response in each case [24,25]. Herein, we report that the ion complexation profile of dye **1** in aqueous environment is characterized by an enhanced selectivity to Hg^2+^ ions, and complex formation leads to changes in its emission spectrum shape and intensity. Compound **1** is demonstrated to penetrate into living cells and can be used for the ratiometric detection of Hg(II) in vivo. The concentration range of Hg^2+^ ion detection in cells (37 nM–1 μM) provided by dye **1** is suitable for analytical applications. As a supporting study useful for the analysis of sensor behavior in biological systems, we also present the investigation of metal cation binding properties of dyad **1** and monochromophoric compounds **2** and **3** (Scheme 1) in an aqueous solution.

## 2. Materials and Methods

### 2.1. Reagents for Spectroscopic Studies

The bis(styryl) dye **1** was obtained by condensation of the formyl derivatives of benzo-15-crown-5 and *N*-phenylazadithia-15-crown-5 ethers with *γ*-picoline and subsequent quaternization of the intermediate styryl(pyridine) species with 1,4-bis(bromomethyl)benzene [24]. The parent mono-chromophoric dyes **2** and **3** were synthesized according to published protocols [26,27]. Acetate buffer solution was prepared with deionized water (18.2 MΩ·cm). Perchlorates of Cu^2+^, Zn^2+^, Pb^2+^, Cd^2+^, Ni^2+^, Fe^2+^, Ca^2+^, and Mg^2+^ were dissolved in MeCN (HPLC grade) and then used in spectroscopic studies. Hg(ClO_4_)_2_ was dissolved in water and stabilized by the addition of 0.5 equivalents (equiv.) of HClO_4_. The exact concentration of Hg(ClO_4_)_2_ was determined by complexometric titration using EDTA and xylenol orange as an indicator. Stock solutions of compounds **1**, **2**, and **3** for the steady-state optical measurements and for the determination of stability constants were prepared with HPLC grade acetonitrile and were of a concentration of 5 × 10^–4^ M. Before collecting the spectra, these solutions (100 μL) were added to acetate buffer solution or pure water (2.5 mL). The MeCN content in the resulting mixtures was 3.8 vol. %.

### 2.2. Steady-State Optical Measurements in Solution

Absorption and fluorescence spectra of the compounds were recorded in air-saturated solutions at ambient temperature with a Cary 300 spectrophotometer (Agilent Technologies, Victoria, Australia) and Eclipse Cary spectrofluorimeter (Agilent Technologies, Victoria, Australia). All measured fluorescence spectra were corrected for the nonuniformity of detector spectral sensitivity. Coumarin 481 in acetonitrile (fluorescence quantum yield *φ*^fl^ = 0.08) [28] was used as a reference for the fluorescence quantum yield measurements.

### 2.3. Determination of Complex Stability Constants

Complex formation of ligands **1** and **3** with Hg^2+^ was studied in aqueous solution by spectrophotometric and spectrofluorometric titration [29,30]. Compound **1** of known concentration in acetate buffer (pH 6.0, 10 mM) was titrated with a solution of mercury (II) perchlorate. After addition of each aliquot of Hg(ClO_4_)_2_ absorption or fluorescence spectrum was recorded, and the stability constants of the complexes were determined using the SPECFIT/32 program (Spectrum Software Associates, West Marlborough, MA, USA). The following equilibria were considered in the fitting of experimental data:

(1) $$L+{\mathrm{Hg}}^{2+}⇄\left(L\right)\cdot {\mathrm{Hg}}^{2+}$$

(2) $$2L+{\mathrm{Hg}}^{2+}⇄{\left(L\right)}_{2}\cdot {\mathrm{Hg}}^{2+}$$

In doing so, it was found that the experimental data corresponded to the theoretical ones if only the Equation (1) was taken into account, and the formation of the complex with composition of 2:1 was assumed to be negligible.

### 2.4. Cells and Their Treatment

Human lung adenocarcinoma cells A549 were obtained from Ivanovsky institute of Virology (Russia) and grown in DMEM-Eagle medium supplemented with L-glutamine (2 mM) and 10% fetal bovine serum (FBS), abbreviated below as a complete medium. Cells were subcultured two times per week (37 °C, 5% CO_2_).

For microscopic experiments, cells were seeded on a round cover glasses placed in 24-well plates and grown for 24 h. Sowing density was 2 × 10^5^ cells per ml. To study cellular accumulation and distribution of **1**–**3** as well as uptake kinetics of **1**, cells were incubated (5–60 min, 37 °C) with **1**–**3** (10 μM) added from 1 mM dimethylsulfoxide (DMSO) solutions in a complete medium, washed twice with Hanks’ solution, and subjected to microscopy analysis. Retention of **1** in cells was studied for cells pre-incubated with **1** (10 μM) for 30 min, placed in the fresh medium (without **1**) for different (0–2.5 h) periods of time, and recorded with confocal laser scanning microscope at the identical parameters of measurements. For the study of intracellular complexation of Hg^2+^ with **1** and **3** or Cu^2+^ and Pb^2+^ with **1**, the cells were pre-incubated (15 min) with Hg(ClO_4_)_2_ at 2–50 μM and Pb[ClO_4_]_2_ or Cu[ClO_4_]_2_ at 2 μM–1 mM. Next, the cells were washed twice with Hanks’ solution, incubated (20 min) with **1** or **3** at 10 μM, washed twice with Hanks’ solution, and subjected to microscopy measurements. It should be mentioned that the used regime of incubation of A549 cells with compound **1** (10 µM) and Hg(ClO_4_)_2_ (2–20 µM) did not induce death of cells as verified by the live/dead cell assay based on differential ability of Hoechst 33,342 and propidium iodide (PI) to penetrate in living and dead cells (for details see Section S5 of Supplementary Materials and Figure S20). Dead cells (15–17%) appeared at 50 µM Hg(ClO_4_)_2_ as confirmed by the Hoechst 33342/PI assay. They were easily recognized due to their round shape and excluded from the measurements.

Cytotoxicity of compound **1** and Hg(ClO_4_)_2_ for A549 cells was additionally estimated after 24 h incubation of cells with **1** or Hg(ClO_4_)_2_ separately, using MTT assay as described in Section S5 of Supplementary Materials. The highest concentration of **1** used in the presented studies (10 μM) did not affect the cell growth (Figure S21b). As for Hg(ClO_4_)_2_, survival of cells decreased at Hg(ClO_4_)_2_ concentrations higher 2.5 μM, and the effect achieved ca. 30% at 20 μM (Figure S21a).

### 2.5. Confocal Microscopy Measurements

Fluorescence microscopy studies of **1**–**3** were performed with the LSM-710 confocal laser scanning microscope (Carl Zeiss AG, Oberkochen, Germany). The confocal fluorescent images were obtained with the α Plan-Apochromat 100×/1.4 oil-immersion objective at 0.3 μm lateral and 1.5 μm axial resolution. Studying the intracellular distribution of **1** and 2 or the kinetics of cellular accumulation of **1** in cells, fluorescence was excited at the 405 nm wavelength, and emission was registered in the 450–730 nm spectral range for **1** or in the 420–650 nm range for **2**. The 488 nm excitation wavelength and the 500–730 nm detection spectral range were used in the studies of intracellular distribution of **3**.

For a ratiometric analysis (intracellular complexation of **1** with Hg^2+^, Cu^2+^, or Pb^2+^), fluorescence was excited at 405 or 488 nm wavelength and recorded simultaneously in the 500–600 and 600–730 nm spectral ranges. Alternatively, fluorescence of **1** was excited at the 405 nm wavelength and registered simultaneously in the 450–550 and 550–730 nm spectral ranges (see Supplementary Information). Intracellular complexation of **3** with Hg^2+^ was studied at the 488 nm excitation wavelength, and emission was registered simultaneously in the 500–600 and 600–730 nm spectral ranges.

Intracellular fluorescence spectra of compounds **1**–**3** were recorded using the spectral mode of confocal image measurements. Fluorescence was excited at 405 nm wavelength and recorded in the 420–730 or 420–650 nm spectral ranges with a spectral resolution of 5 nm for **1** or **2**, respectively. Fluorescence was excited at 488 nm wavelength and recorded in the 500–725 nm spectral range with a spectral resolution of 5 nm for **1** and **3**.

## 3. Results and Discussion

### 3.1. Spectral Characteristics of the Compounds

**Figure 1:**
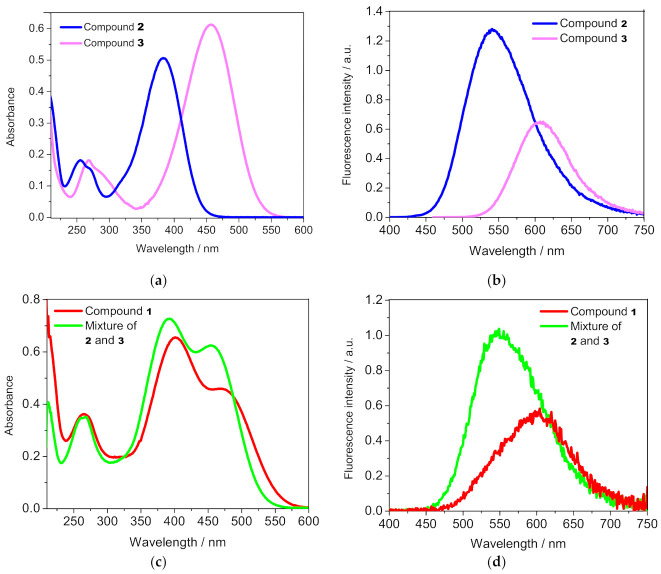
Absorption (**a**,**c**) and fluorescence (**b**,**d**) spectra of compounds **1** (**c**,**d**), **2** (**a**,**b**), **3** (**a**,**b**), and equimolar mixture of **2** and **3** (**c**,**d**) in water at pH 6.0 (acetate buffer, 10 mM). Concentrations of all compounds 20 µM. Excitation wavelengths $\lambda_{\mathrm{ex}}$ were 450 nm for **3** and 380 nm for **1**, **2**, and mixture of **2** and **3**.

Spectroscopic and Hg^2+^-binding properties of probe **1** and its constituents **2** and **3** were studied in aqueous solution (Figure 1, Table 1). All experiments were carried out at pH 6.0 (acetate buffer, 0.01 M), which, on the one hand, is rather close to slightly acidic environment inside A549 cells [31] (these cells were used in cellular experiments below), and on the other hand, excludes protonation at the azacrown ether nitrogen atom and related spectral shifts. Protonation at nitrogen atom of *N*-phenylazadithia-15-crown-5 ether receptor in water solution occurs at pH values lower than 4.0, as it has been shown for the previously synthesized 4-amino-*N*-aryl-1,8-naphthalimide [32,33], where the *N*-phenylazadithia-15-crown-5 ether receptor is present as an *N*-aryl group and not conjugated with the chromophoric part of the molecule. In this sense, probe **1** should be even less basic due to delocalization of the amine nitrogen lone pair electrons into the π-system of positively charged styryl(pyridinium) fragment. As Figure 1a,b shows, maxima of absorption and emission spectra of compound **2** are at 380 and 540 nm, respectively, whereas corresponding maxima of **3** are centered at 460 and 610 nm. From these observations, one can notice that considerable part of the emission spectrum of **2** overlaps with the long-wavelength absorption band of **3** (see Figure S2 for the graphical representation). Regarding dye **1**, such an overlap means that RET from the excited donor *O*-chromophore (benzo-15-crown-5-containing fragment) to acceptor *N*-chromophore (azadithia-15-crown-5-containing fragment) could proceed effectively.

Absorption spectrum of compound **1** (Figure 1c) contains two peaks in the long wavelength region at 402 and 469 nm, which are characteristic of both individual dyes **2** and **3**. Some red shift (10–15 nm) in the peak positions on going from equimolar mixture of **2** and **3** to dye **1** can be explained by the increased polarization of the chromophores and, hence, the more pronounced ICT interaction, resulting from proximity of two positive charges in the structure of **1**.

Upon excitation by 380 nm light, which is mostly absorbed by *O*-chromophore, equimolar mixture of **2** and **3** exhibited the emission band at 548 nm (Figure 1d), similar to that in the spectrum of **2** (Figure 1b). The spectrum of **1** recorded under the same conditions consisted of the red-shifted signal ($\lambda_{\max}^{\mathrm{fl}}$ 603 nm) corresponding to the *N*-chromophore fluorescence. This result indicates preservation of RET in the compound **1** in slightly acidic aqueous environment. Noteworthily, comparison of the spectra of **1** and **3** presented in Figure 1b,d shows that the short wavelength part of the emission band of **1** (Figure 1d) is broadened to some extent by the residual fluorescence of the donor. Using the emission intensities at 490 nm where the acceptor chromophore does not emit light (see Figure 1b) for compound **1** (I) and equimolar mixture of **2** and **3** ($I_{0}$), the energy transfer efficiency was calculated to be as high as 0.84 (84%) according to the Equation (3) [34]:

(3) $$\Phi_{\mathrm{RET}}=1-\frac{I}{I_{0}}$$

A rather close value of $\Phi_{\mathrm{RET}}$ (91%) was obtained using the theoretical calculations by the Fӧrster model [34] (for details see Supplementary Information).

### 3.2. Complexation with Metal Cations in Solution

**Figure 2:**
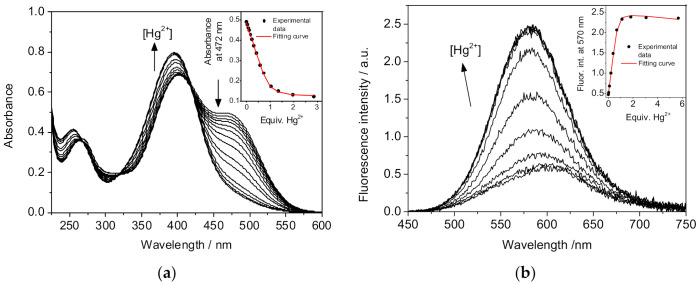
Changes in the absorption (**a**) and fluorescence (**b**) spectra of compound **1** (2 × 10^–5^ M) upon addition of aliquots of Hg[ClO_4_]_2_ in water at pH 6.0 (acetate buffer, 0.01M). Excitation wavelength was 380 nm. The upper inserts show the absorbance at 472 nm (**a**) or fluorescence intensity at 570 nm (**b**) versus equivalents of Hg^2+^ added.

Next, we proceeded to the complex formation experiments. In contrast to previously reported data obtained in acetonitrile [24,25], addition of Cu^2+^, Zn^2+^, Pb^2+^, Cd^2+^, Ni^2+^, Fe^2+^, Ca^2+^, and Mg^2+^ cations did not result in significant changes in the absorption and emission spectra of probe **1** in aqueous solution (Figures S5–S12). In the same conditions, an addition of gradual amounts of Hg^2+^ decreased dramatically the absorbance of **1** at 470 nm with a concomitant growth of the intensity of the short wavelength band (Figure 2a). The Hg^2+^-induced changes in the fluorescence spectrum of **1** obtained under the excitation at 380 nm consisted in an enhancement of the emission signal, which was also found to be blue shifted with respect to the parent position (Figure 2b). The latter observation allows one to consider dye **1** as a ratiometric probe (for more clarity see the influence of Hg^2+^ on the normalized emission spectrum, Figure S13). The spectra obtained at different concentrations of Hg^2+^ cation were consistent with the formation of the complex with 1:1 metal to ligand ratio in aqueous solution and were applied for the calculation of the complex stability constant (see Table 1 and Section 2.3 for details). The competition experiments were also conducted (Figure S14). In a typical procedure, the emission spectrum was measured before and after the addition of other ions (5 equiv.) to the solution of **1** (2·10^–5^ M) containing Hg^2+^ (1 equiv.). It was found that the presence of Cu^2+^, Zn^2+^, Pb^2+^, Cd^2+^, Ni^2+^, Fe^2+^, Ca^2+^, and Mg^2+^ did not interfere with Hg^2+^ sensing.

To explain the observed spectral effects, we further evaluated the influence of Hg^2+^ addition on the spectroscopic properties of the monochromophoric derivatives **2** and **3**. In the case of oxacrown compound **2**, no changes in the position and intensity of the long wavelength absorption and emission bands were found (Figure S17), indicating that the energy donor chromophore in **1** is not involved in Hg^2+^ coordination. The presence of benzo-15-crown-5 ether group in the structure of probe **1** is supposed to increase its solubility in aqueous medium. As we have also found, this group is capable to bind with Na^+^ and K^+^ cations at concentrations higher than 1 mM in water (see the changes in the absorption and fluorescence spectra of **1** upon addition of Na^+^ and K^+^, Figures S15 and S16). However, sensing of Hg^2+^ with **1** apparently copes with such complexation as the 0.01 M acetate buffer solution (the medium used in complex formation studies) was prepared using CH_3_COONa and also contained high Na^+^ concentration.

Azacrown ether derivative **3** demonstrated a pronounced (77 nm) blue shift in the absorption spectrum (see Table 1 and Figure S18a) with a new band of the complex (3)·Hg^2+^ arising at 380 nm and isosbestic point at 408 nm. Under the excitation at 405 nm, where the absorption values of the ligand **3** and (**3**)·Hg^2+^ are rather close, the emission spectrum of **3** showed an increase in the intensity and a shift of $\lambda_{\max}^{\mathrm{fl}}$ to a short wavelength region (Figure S18b). Based on the analysis of the presented data, the fluorescence enhancement can be ascribed to a higher fluorescence quantum yield of (**3**)·Hg^2+^ complex (Table 1), whereas the blue shift could be a result of a reduced ICT interaction between the donor azacrown ether nitrogen atom coordinated with the cation and the acceptor *N*-alkylpyridinium moiety.

Bearing in mind these points, one can assume that the changes in the absorption spectrum of **1** in the presence of Hg[ClO_4_]_2_ are related only with the hypsochromic shift of the *N*-chromophore ICT band caused by the coordination of azadithia-15-crown-5 ether receptor with mercury (II) cation. The nature of the fluorescence response of **1**, however, appears to be more intricate. Excitation wavelength 380 nm used for spectrofluorometric titration corresponds to the absorption maximum of the complex of *N*-chromophore with Hg^2+^ (Table 1). This means that the observed fluorescence enhancement (Figure 2b) is in part related to the direct excitation of the *N*-chromophore fragment in complex (**1**)·Hg^2+^, the concentration of which increases during the titration experiment. At the same time, the wavelength 380 nm is close to $\lambda_{\max}^{\mathrm{abs}}$ of the oxacrown derivative 2 (Table 1), which allows us to assume some contribution from the *O*-chromophore fluorescence in the emission band of (**1**)·Hg^2+^. Indeed, the hypsochromic shift observed for compound **3** upon binding with Hg^2+^ leads to a decrease in the overlap of its absorption band with emission spectrum of **2** (Figure S2). According to the calculations by the Förster theory (see Supplementary Information), this could reduce the $\Phi_{\mathrm{RET}}$ value from 84% (for the free ligand **1**, see above) to 31% (for (**1**)·Hg^2+^). Hence, a considerable part of the excited donor fragments in (**1**)·Hg^2+^ may deactivate radiatively, thereby contributing to the overall spectrum. In contrast to the free ligand **1**, the short wavelength part of (**1**)·Hg^2+^ fluorescence band was not broadened, because of the close $\lambda_{\max}^{\mathrm{fl}}$ values for *O*-chromophore and complexed *N*-chromophore emission (compare $\lambda_{\max}^{\mathrm{fl}}$ for compound **2** and (**1**)·Hg^2+^ in Table 1).

### 3.3. Cellular Imaging of the Studied Compounds

**Figure 3:**
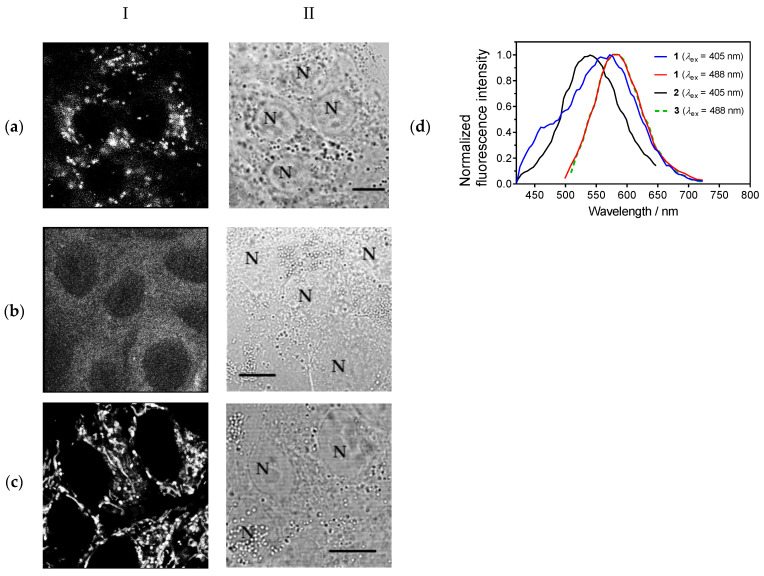
Confocal fluorescence images (**a**–**c**) and fluorescence spectra (**d**) of **1**–**3** in A549 cells. (**a**–**c**) Typical intracellular distributions (column I) of **1** (**a**), **2** (**b**), and **3** (**c**) and corresponding transmitted light images of cells (column II). Bar represents 10 μm. N marks a nucleus. Cells were incubated with **1**–**3** (10 μM) for 30 min. (**d**) Typical intracellular fluorescence spectra of **1**, **2**, and **3** normalized to the maximum. At λ_ex_ = 488 nm, spectra of **1** and **3** coincide in a shape.

We investigated applicability of **1** for the fluorescent imaging of Hg^2+^ in living cells and utilized compounds **2** and **3** to clarify intracellular properties of **1**. Using confocal laser scanning microscopy (CLSM), it was revealed that **1**–**3** penetrated in the human lung adenocarcinoma A549 cells and accumulated in cytoplasm (Figure 3). At the optimized excitation and detection conditions, compounds **1** and **3** fluoresced brightly in cells, while cellular fluorescence of **2** was weak. The patterns of intracellular distribution of **1**–**3** were found to be different. Accumulation in vesicular structures of a submicron size and diffuse staining of cytoplasm were observed for **1** (Figure 3a). Compound **2** demonstrated diffuse staining of cytoplasm (Figure 3b). Compound **3** accumulated in elongated structures forming a complex network (Figure 3c), whose morphology is typical for mitochondria.

To clarify an origin of organelles accumulating compounds **1** and **3**, a colocalization analysis of vital fluorescent probes for particular organelles and the studied compounds was performed. A study of colocalization of **3** and rhodamine 123, a selective fluorescent probe of mitochondria, confirmed mitochondrial accumulation of **3** in A549 cells (Figure S22). The vesicles stained with **1** were demonstrated to be different from lipid droplets, i.e., cellular organelles that store neutral lipids (Figure S23). Using chlorin *e*_6_ derivative (see Figure S24 for the structure), which was previously shown to accumulate in lysosomes [35], it was revealed that compound **1** is localized in lysosomes (Figure S25).

Intracellular fluorescence spectra of compounds **1**–**3** were analyzed using the spectral mode of confocal image measurements. At excitation wavelength *λ*_ex_ = 488 nm, compounds **1** and **3** have very similar intracellular spectra with a maximum at ca. 584 nm (Figure 3d). Intracellular fluorescence spectrum of **2** (*λ*_ex_ = 405 nm) has a maximum at ca. 543 nm. Fluorescence spectra of compounds **1** (*λ*_ex_ = 488 nm), **2**, and **3** were not noticeably changed in a shape in different areas of cells, but varied in intensity. Comparison with the fluorescence spectra of **1**–**3** measured in an aqueous solution (Figure 1, Table 1) shows that intracellular spectrum of **2** has the same maximum, while spectrum maxima of **1** and **3** are blue-shifted by ca. 30 nm, probably due to interactions of **1** and **3** with some cellular molecules. Similar blue shift of the ICT fluorescence band has been observed for the previously described 4-*N*,*N*-dimethylaminostyryl derivative in A549 cells [36].

Intracellular fluorescence spectrum of **1** excited at 405 nm is very wide, centered at ca. 570 nm, and exhibit a shoulder in the 420–480 nm spectral range (Figure 3d). The analysis shows that this spectrum can be described as a superposition of spectra of **2** and **3** and a residual spectrum having a maximum at 459 nm (Figure S26). Relative contribution of these three components into the spectrum of **1** varies in different areas of a cell (Figure S26). In the 500–700 nm spectral range, fluorescence of **1** is a superposition of oxacrown and azacrown ether moiety emissions with comparable contributions. This could be explained by an incomplete energy transfer in the compound **1** (84%, see Section 3.1) along with the relatively high emission quantum yield of the donor chromophore: $\varphi^{\mathrm{fl}}$ value for **2** is 2.4 times higher compared with that for **3** (Table 1). Reasonably, some changes in RET efficiency and/or fluorescence quantum yields of the photoactive fragments (fluorescence quantum yields of styryl dyes exhibiting the properties of ICT-fluorophores are very sensitive to the polarity of the medium [37,38,39]) in compound **1** may occur upon going from aqueous solution to cellular medium. For instance, RET can be hampered as a result of a specific conformation change (a decrease in RET can occur, for example, if intermolecular interactions of **1** with proteins and/or lipids lead to a fixed (or restricted) orientation of donor relative to acceptor moiety; considerably reduced RET is predicted by the Fӧrster theory for the orientations, when an angle between the vectors of donor and acceptor transition dipole moments is close to 90° [34]) or a decrease in the extent of overlap between the donor emission and acceptor absorption spectrum. The “residual” component at 459 nm is not related to cellular autofluorescence, because it is absent in non-treated cells and cells treated with **2** or **3**. Probably, the 459 nm component is a signature of particular complexes formed by **1** in cells, and this fluorescence is emitted by oxacrown moiety.

Measurement of kinetics of 1 internalization revealed that intracellular accumulation of **1** achieved saturation after 15 min of incubation of A549 cells with **1**, and time of half-accumulation was 4.0 ± 0.2 min (Figure S27a). Accordingly, all the measurements of interactions between **1** and Hg^2+^ in cells were performed after 20 min incubation of cells with compound **1**.

Compound **1** is hydrophobic, and mechanism of its intracellular penetration is supposed to be related (at least partially) to passive diffusion through plasma membrane followed by accumulation and trapping in lysosomes. It is indirectly confirmed by appearance of diffuse distribution of **1** in cytoplasm and weak staining of lysosomes 5 min after the beginning of cell incubation with dye **1** (Figure S28a). In accordance with our supposition, further incubation of cells with **1** is accompanied by an increase in lysosome accumulation of **1** as compared to its diffuse distribution (Figure S28a). One cannot exclude that endocytosis also contributes to internalization of **1**, but the study of mechanisms of cellular transport of **1** is beyond the scope of this paper and requires special research.

Retention of **1** in cells was found to be long. A decrease in intensity of intracellular fluorescence of **1** or changes in the pattern of its intracellular distribution were not observed during 2.5 h after removal of **1** from a culture medium (Figure S27b–e).

**Figure 4:**
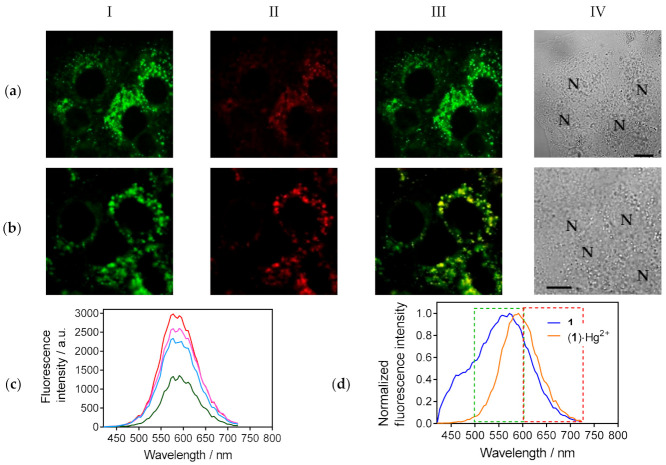
Confocal fluorescence images (**a**,**b**) and fluorescence spectra (**c**,**d**) of **1** and (**1**)·Hg^2+^ in A549 cells. (Row **a**) Cells were incubated with **1** (10 μM) for 20 min. (Row **b**) Cells were pre-incubated with 20 μM Hg(ClO_4_)_2_ for 15 min, washed twice with Hanks’ solution, and incubated with **1** (10 μM) for 20 min. Images of intracellular fluorescence were measured in the 500–600 nm (column I) and 600–730 nm (column II) spectral ranges at *λ*_ex_ = 405 nm. Merged fluorescent images are shown in column III. Transmitted light images of cells are shown in column IV. Bar represents 10 μm. N marks a nucleus. (**c**) Fluorescence spectra of **1** from different regions of cells pre-incubated with Hg(ClO_4_)_2_. (**d**) Typical normalized intracellular fluorescence spectra of **1** and (**1**)·Hg^2+^. Green and red rectangles show two spectral regions (500–600 and 600–730 nm) selected for acquisition of images shown in columns I and II.

Pre-incubation of cells with Hg(ClO_4_)_2_ did not change a qualitative pattern of intracellular distribution of **1** (Figure 4, Figure S29), but resulted in considerable changes in intracellular fluorescence spectra of **1** excited at *λ*_ex_ = 405 nm, thus indicating formation of (**1**)·Hg^2+^ complexes (Figure 4c,d). At a high Hg^2+^ concentration, intracellular fluorescence spectrum of **1** (*λ*_ex_ = 405 nm) becomes narrow and shows a maximum at 590 nm. At *λ*_ex_ = 488 nm, intracellular spectrum of (**1**)·Hg^2+^ is slightly red-shifted as compared to intracellular spectrum of **1** (*λ*_ex_ = 488 nm), has a maximum at 590 nm (Figure S29c), and coincides in shape with the spectra of (**1**)·Hg^2+^ complexes excited at *λ*_ex_ = 405 nm. Such changes in the emission band shape are consistent with the spectrofluorometric titration data obtained in aqueous solution (Figure 2b). As mentioned above (Section 3.2), the broadening of the short wavelength part of the emission spectrum was observed for the free ligand **1** but not for the complex (**1**)·Hg^2+^. In addition, 4.8 times fluorescence enhancement occurred at 580 nm (Figure 2b) due to Hg^2+^-induced increase in the acceptor chromophore quantum yield (compare $\varphi^{\mathrm{fl}}$ values for **3** and (**3**)∙Hg^2+^, see Table 1). Similar increase in the fluorescence intensity (4.3±0.3 times (calculated as the ratio of integral emission intensity in 600–730 nm spectral range after the addition of 50 μM of Hg^2+^ to cells to that in the absence of Hg^2+^)) also was found upon coordination of Hg^2+^ with **1** in cells. Obviously, the enhanced emission from the complexed *N*-chromophore in solution and in cells would lead to a low contribution of *O*-chromophore fluorescence to the overall spectrum.

**Figure 5:**
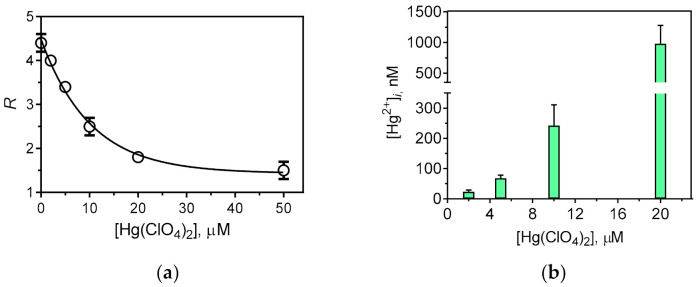
Detection of intracellular Hg^2+^ ions using a ratiometric approach with compound **1**. (**a**) Dependence of the ratio *R* of fluorescence intensities *I* (500–600 nm)/*I* (600–730 nm) on the concentration of Hg(ClO_4_)_2_ added to cells. Data were averaged over 20 cells in each measurement and presented as a mean ± SD. (**b**) Estimation of intracellular concentration of Hg^2+^ ions ([Hg^2+^]*_i_*) at different concentrations of Hg(ClO_4_)_2_ added to cells. Cells were pre-incubated with different concentrations of Hg(ClO_4_)_2_ for 15 min, washed twice with Hanks’ solution, incubated with **1** (10 μM) for 20 min, and measured with CLSM.

The observed differences in intracellular fluorescence spectra of **1** and (**1**)·Hg^2+^ at *λ*_ex_ = 405 nm can be used for a ratiometric fluorescent detection of Hg^2+^ in cells using compound **1**. For example, measuring fluorescent images of **1** within cells in the 500–600 nm and 600–730 nm spectral ranges simultaneously at *λ*_ex_ = 405 nm, one can see that relative fluorescence intensities in these images are noticeably different in the presence and absence of Hg^2+^ ions (Figure 4a,b). The ratio *R* of the fluorescence intensities *I* (500–600 nm)/*I* (600–730 nm) calculated for and averaged over a sampling of cells (*n* = 20) is equal to 4.4 ± 0.2 in the absence of Hg^2+^ ions and 1.43 ± 0.10 at the saturation of Hg^2+^ binding to **1**. The ratio *R* decreases as a function of Hg(ClO_4_)_2_ concentration added to cells (Figure 5a), indicating that *R* is sensitive to intracellular concentration of Hg^2+^. А choice of two spectral ranges for an intracellular ratiometric detection of Hg^2+^ with compound **1** can be made in different ways. Alternatively, the 550–730 and 450–550 nm spectral ranges can be selected (Figure S33). In the last case, the ratio of fluorescence intensities *I* (550–730 nm)/*I* (450–550 nm) varies from 1.7 ± 0.2 in the absence of Hg^2 +^ ions to 4.8 ± 0.3 for the (**1**)·Hg^2+^ complex, and complexation with Hg^2+^ is clearly observed in the recorded images (Figure S33).

It should be mentioned that at the direct excitation of acceptor moiety (for example, with *λ*_ex_ = 488 nm), intracellular fluorescence spectra of **1** and (**1**)·Hg^2+^ are close in shape and maxima (Figure S29), and (**1**)·Hg^2+^ complex formation is mainly manifested by an increase in fluorescence intensity. At the saturation of Hg^2+^ binding to **1**, intracellular intensity of fluorescence increases by 4.1 ± 0.1 times as compared to that of free **1**. Therefore, alterations in intracellular fluorescence intensity of **1** at *λ*_ex_ = 488 nm can be used (with some limitations) as an indicator of changes in intracellular concentration of Hg^2+^, when it is known that Hg^2+^ ions are present in cells. In contrast, ratiometric measurements with **1** at *λ*_ex_ = 405 nm are applicable both for detection of Hg^2+^ ions in cells and measurement of changes in their concentration.

Because of weak intracellular fluorescence intensity and absence of changes in a spectrum shape, compound **3** was concluded to be not suitable for intracellular analysis of Hg^2+^ ions (Figure S30). Absence of changes in intracellular fluorescence spectra of **3** was observed at different incubation time of A549 cells with Hg^2+^ and 3 (5–30 min) in the wide range of concentrations of Hg(ClO_4_)_2_ (20–100 μM) and **3** (5–30 μM). Definitely, spectral properties of **3** in cells differ considerably from those in an aqueous solution (Figure S18b), and reasons for these differences require a special study. One of the probable reasons is a basic pH (pH = 8.0) in mitochondria [40], where compound **3** is accumulated. As shown earlier [41,42], *N*-phenylazadithia-15-crown-5 ether receptor does not form stable complexes with Hg^2+^ at pH > 7.0 in solution, because mercury (II) ions are completely hydrolyzed at neutral and basic pH and exist in the form of Hg(OH)_2_ [43].

In order to verify selectivity of intracellular response of **1** to Hg^2+^ ions, A549 cells were pre-incubated with Cu^2+^ or Pb^2+^ ions in the concentration range of 2–1000 µM, and further incubated with **1** (10 µM). No changes in either intracellular fluorescence spectra (including intensity) or a characteristic pattern of intracellular distribution of **1** were observed in the studied range of Cu^2+^ and Pb^2+^ concentrations (Figures S31 and S32). This result is in line with inability of **1** to form complexes with Cu^2+^ and Pb^2+^ in aqueous solution (see Supplementary Information, Figures S5 and S6), and let us to conclude that compound **1** is promising for selective detection of Hg^2+^ ions in living cells using a ratiometric approach.

Following the formalism proposed elsewhere [44], the ratiometric measurements with **1** can be used to estimate intracellular concentrations of Hg^2+^ ions ([Hg^2+^]*_i_*), as shown in the Equation (4):

(4) $${\left[{\mathrm{Hg}}^{2+}\right]}_{i}=K_{d}\times Q\times \frac{R_{max}-R}{R-R_{min}}\;,$$

where *K_d_* is the dissociation constant of (**1**)·Hg^2+^ complex calculated from lg *K* (Table 1) and equal to 0.56 ± 0.15 µM (Calculated using *K* value obtained in spectrophotometric titration experiment at pH 6.0. It should be noted that localization of probe **1** in lysosomes where the pH is more acidic (4.5–4.8) [45] would not impair the [Hg^2+^]*_i_* estimation. The lg *K* values for (**1**)·Hg^2+^ complex obtained in spectrophotometric and spectrofluorometric titration experiments in 1 × 10^–2^ M acetate buffer at pH 4.5 (6.43 ± 0.15 and 6.46 ± 0.19, respectively, see Figure S19 for titration data) were very close to those calculated at pH 6.0 (6.27 ± 0.12 and 6.24 ± 0.11, respectively, Table 1)), *R_max_* and *R_min_* are the *R* values for the free ligand **1** (*R_max_* = 4.4 ± 0.2), and for the complex (**1**)·Hg^2+^ (*R_min_* = 1.43 ± 0.10) in cells found using the plot in Figure 5a, *Q* is a ratio of intracellular fluorescence intensity of the free ligand **1** to the intensity of (**1**)·Hg^2+^ in the 600–730 nm range (*Q* = 0.23 ± 0.03).

It should be noted that the calculations of [Hg^2+^]*_i_* by the formula (4) allow quantification of only equilibrium concentration of the “free” mercury (II) ions. If one needs to find total amount of Hg(II) inside cells (including Hg(II) trapped in stable complexes with some species like thiol compounds), some other spectroscopic techniques such as atomic absorption spectroscopy [46] should be used. Regarding complexation with thiols, we have found that addition of a slight excess (5 equiv.) of cysteine (Cys), a model thiol compound, to a solution of complex (**1**)·Hg^2+^ (obtained by mixing of 10 μM dye **1** and 10 μM Hg(ClO_4_)_2_) causes a decrease in the emission intensity and changes in the absorption band shape, which are consistent with the complete release of the free ligand **1** from the complex (compare the spectrum of **1** with that of the mixture of 1 equiv. **1**, 1 equiv. Hg(ClO_4_)_2_, and 5 equiv. Cys, Figure S34). From the spectral changes shown in Figure S34b and the known stability constant of the complex (**1**)·Hg^2+^ (lg *K* = 6.27 ± 0.12, Table 1), we estimated the lg *K* value for the complex of Hg^2+^ with cysteine ((Cys)·Hg^2+^) to be as high as 6.60 ± 0.20. Thus, (Cys)·Hg^2+^ and (**1**)·Hg^2+^ demonstrate comparable stability. Based on this observation, we can conclude that thiol compounds present in cells (glutathione and thiol containing proteins and peptides) can bind efficiently Hg^2+^ ions, thereby decreasing concentration of the free Hg^2+^ available for complexation with **1**.

Results of [Hg^2+^]*_i_* estimation on the basis of *R* values measured at different concentrations of Hg(ClO_4_)_2_ added to cells are shown in Figure 5b. The dependence of *R* on intracellular concentration of Hg^2+^ in the 0–60 nM range demonstrated a good linearity with a correlation coefficient of 0.99 (Figure S35). From the slope of this linear dependence (*r*) and standard deviation of the ratio *R* (s), the lower detection limit for Hg^2+^ in cells ($C_{\mathrm{DL}}$) was found to be 37 nM, according to the Equation (5) [47], which is comparable with the other fluorescent Hg^2+^ chemosensors [33,48].

(5) $$C_{\mathrm{DL}}=\frac{3s}{r}$$

As it can be seen from Figure 5a, an increase of Hg(ClO_4_)_2_ concentration in extracellular medium higher than 20 μM does not result in significant changes of the ratio *R*. Furthermore, the changes of *R* at 20–50 μM Hg(ClO_4_)_2_ are comparable with the measurement accuracy. This means that the upper limit of Hg^2+^ detection inside cells with probe **1** corresponds to the presence of approximately 20 μM of mercury (II) ions in the extracellular medium and, hence, appears to be 1 μM (see Figure 5b). Thus, compound **1** allows quantitative detection of Hg^2+^ ions inside cells in the 37 nM–1 µM concentration range. Varying concentration of compound **1** added to cells the range of quantitative Hg^2+^ ion detection could be slightly extended.

Sensor **1** is not toxic to cells at the concentration of 15 μM or less for 24 h incubation (Figure S21b), and its retention in cells is rather long (Figure S27b–e) that make this probe suitable both for quantitative detection of Hg^2+^ in cells and for long-term studies of Hg^2+^ interactions with living cells. To demonstrate it, we have used sensor **1** for evaluation of changes in intracellular concentration of Hg^2+^ as a function of time after removal of Hg(ClO_4_)_2_ from extracellular medium (Figure S36). It was found that in 30 min after removal of extracellular 10 μM Hg(ClO_4_)_2_, intracellular concentration of Hg^2+^ was measured to be 226 ± 30 nM and decreased during next 30 min to 120 ± 20 nM (Figure S36f), most probably because of Hg^2+^ efflux from cells. Interestingly, during the next 2.5 h intracellular concentration of Hg^2+^ increased to 186 ± 30 nM (Figure S36f), which can be explained by slow release of previously bound Hg(II) ions from intracellular depot.

Using Equation (4) and the plot of *R* against [Hg(ClO_4_)_2_] (Figure 5a), one can estimate that the lower detection limit of intracellular Hg^2+^ (37 nM) is achieved when extracellular concentration of [Hg(ClO_4_)_2_] is as low as 2.8 μM. The estimated value seems to be relevant in terms of sensor applications. For example, fish consumption is known to be the main contributor to the maximal allowable daily intake of Hg for humans. Food guidelines suggest the total mercury content of fish flesh for human consumption should generally not exceed 0.5 μg/g wet weight (or 2.5 μM) [49].

## 4. Conclusions

We have shown that bis(styryl) dye **1** can be applied for the visualization and ratiometric fluorescent measurement of Hg^2+^ concentration in living cells with good selectivity and sensitivity. Optical response of compound **1** includes an enhancement of emission intensity with a concomitant change in the shape of fluorescence spectrum and can be rationalized in terms of RET and ICT sensing mechanisms. Presented results allow us to conclude that compound **1** can find applications in the studies of the role and transformations of mercury (II) cation in complex biological systems.

## Figures and Tables

**Table 1:** Spectral characteristics of compounds **1**, **2**, and **3** and stability constants of their complexes*^a^* with Hg^2+^ in water at pH 6.0 (10 mM acetate buffer) *^a^*.

Species	$\lambda_{max}^{abs}/\mathrm{nm}$	$\varepsilon_{\lambda }/M^{-1}\cdot {\mathrm{cm}}^{-1}$	$\lambda_{max}^{fl}$ $(\lambda_{ex})/\mathrm{nm}$	$\varphi^{fl}$	lgK	
					Spectrophotometric Titration	Spectrofluorometric Titration
**1**	402; 469	32,700; 22,950	603 (380)	0.015	–	–
(**1**)·Hg^2+^	395	40,970	581 (380)	0.041	6.27 ± 0.12	6.24 ± 0.11
**2**	384	25,300	541 (380)	0.029	–	–
**3**	457	30,650	607 (405)	0.012	–	–
(**3**)·Hg^2+^	380	18,390	571 (405)	0.052	6.03 ± 0.06	6.05 ± 0.06

*^a^* Absorption ($\lambda_{\max}^{\mathrm{abs}}$, $\varepsilon_{\lambda }$) and emission ($\lambda_{\max}^{\mathrm{fl}}$, $\varphi^{\mathrm{fl}}$) characterisctics of the complexes were found from their absorption and emission spectra calculated using SPECFIT/32 program (see Figures S3 and S4).

## Data Availability

Not applicable.

## Supplementary Materials

The following are available online at https://www.mdpi.com/1424-8220/21/2/470/s1, Figures S1–S36, calculations of RET efficiency in ligand **1** and complex (**1**)∙Hg^2+^, experimental details and results concerning cell viability studies and studies on intracellular localization of **1** and **3**.
