# Supplementary material for: Ratiometric Detection of Mercury (II) Ions in Living Cells Using Fluorescent Probe Based on Bis(styryl) Dye and Azadithia-15-Crown-5 Ether Receptor
Source: Sensors (Basel). 2021 Jan 11;21(2):470. doi: 10.3390/s21020470 (PMC7826577; doi:10.3390/s21020470)
Supplement: Supplementary file 1 [file sensors-21-00470-s001.pdf]

Supplementary Materials

Ratiometric Detection of Mercury (II) Ions in Living Cells Using Fluorescent Probe Based on Bis(styryl) Dye and Azadithia-15-Crown-5 Ether Receptor

Pavel A. Panchenko [1,2,*], Anastasija V. Efremenko [3,4], Alexey V. Feofanov [3,4], Mariya A. Ustimova [1], Yuri V. Fedorov [1] and Olga A. Fedorova [1,2]

[1] Laboratory of Photoactive Supramolecular systems, A.N.Nesmeyanov Institute of Organoelement Compounds of Russian Academy of Sciences (INEOS RAS), 119991 Moscow, Russia; ustimova.maria@yandex.ru (M.A.U.); fedorov@ineos.ac.ru (Y.V.F.); fedorova@ineos.ac.ru (O.A.F.)
[2] Department of Technology of Fine Organic Synthesis and Chemistry of Dyes, Dmitry Mendeleev University of Chemical Technology of Russia, 125047 Moscow, Russia
[3] Biological Faculty, Lomonosov Moscow State University, 119992 Moscow, Russia; aefr@mail.ru (A.V.E.); avfeofanov@yandex.ru (A.V.F.)
[4] Laboratory of Optical Microscopy and Spectroscopy, Shemyakin-Ovchinnikov Institute of Bioorganic Chemistry of Russian Academy of Sciences, 117997 Moscow, Russia
* Correspondence: pavel@ineos.ac.ru, Tel.: +7-905-525-07-93

CONTENTS

1. Calculation of RET efficiency in the ligand **1** and (**1**)·Hg^{2+} complex………….……………………..…S2
2. Calculated absorption and emission spectra of ligands **1** and **3**and complexes (**1**)·Hg^{2+} and (**3**)·Hg^{2+}…………………………………………………………….……………………….…..….S4
3. Influence of metal cations on the absorption and emission spectra of compound **1**……………………………………………………………………………….……………..……S5
4. Spectrophotometric and spectrofluorometric titration of ligands **1**, **2**, and **3** with Hg^{2+} S9
5. Cell viability studies…………………….……………………………………………….…….……….....S10
6. Intracellular localization of compounds **3** and **1**………………….……………………..…………..S12
7. Intracellular fluorescence spectra of **1** in A549 cells…………….……………………………………...S15
8. Kinetics of intracellular accumulation and retention of **1**………..……………………….………S16
9. Probing interactions of **1** and **3** with Hg^{2+} in cells……………………..…………………………………...S17
10. Interactions of **1** with Cu^{2+} and Pb^{2+} in living cells…………………………………………..…….…S19
11. Alternative choice of spectral ranges for ratiometric detection of Hg^{2+} in cells………………………...S21
12. Influence of cysteine addition on the absorption and emission spectrum of (**1**)·Hg^{2+}…………………………………………………………………………...………….……..S22
13. Plot of fluorescence intensity versus increasing concentrations of Hg^{2+} in cells………………………..S23
14. Detection of Hg^{2+} concentration changes in cells………………………………………………….….S23
15. References……..………………...……...………………………………………………………….S24

1. Calculation of RET Efficiency in the Ligand 1 and (1)·Hg^{2+} Complex

Resonance energy transfer efficiency Φ_{RET} in the ligand **1** and (**1**)·Hg^{2+} complex was calculated by the Equation (S1) [1]:

$$\Phi_{\mathrm{RET}} = \frac{R_0^6}{R_0^6 + r^6} \quad \text{(S1)}$$

where r is the distance between the donor and the acceptor, and R_0 is the critical Förster radius at which 50% of the excited donors deactivate through the RET pathway (i.e., $\Phi_{\mathrm{RET}} = 0.5$).

The value r was found from the optimized ground state geometry of **1** (Figure S1, r = 15.3 Å). The three dimensional structure of **1** was built with MOPAC 2016 program package using PM7 semiempirical method [2]. The calculations were performed at optimized geometries, which reached gradient variations less than 0.01 kcal/mol. The solvent effect was included in geometry optimizations following the "COnductorlike Screening Model" (COSMO) implemented in MOPAC. A dielectric constant of ε = 80 and a refraction index of solvent (n) such that n^2 = 2 were used. For the calculation of Φ_{RET} in the complex (**1**)·Hg^{2+} the same r value (15.3 Å) was used.

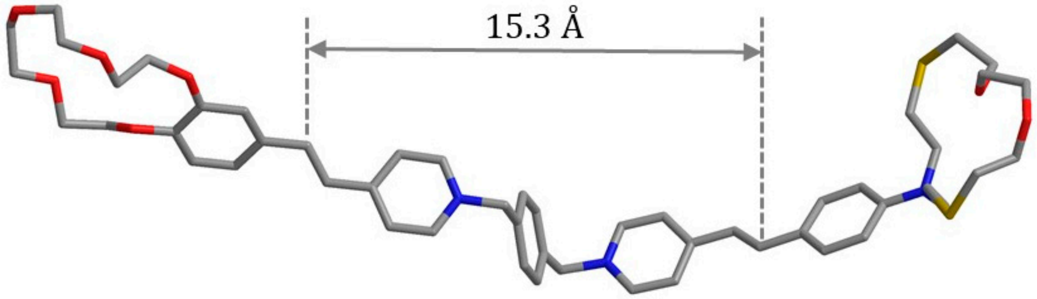

Figure S1. Optimized ground state geometry of the compound **1**.

R_0 was calculated according to the Equation (S2) [1]:

$$R_0^6 = 8.79 \times 10^{-5}\left[\kappa^2 \times n^{-4} \times \varphi_{D,0}^{\mathrm{fl}} \times J(\lambda)\right] \quad \text{(S2)}$$

where κ^2 is an orientation factor that depends on the mutual disposition of the donor and acceptor transition dipole moments, $\varphi_{D,0}^{\mathrm{fl}}$ is the fluorescence quantum yield of the donor in the absence of the acceptor, n is the refractive index of the solvent, N_A is Avogadro constant, and $J(\lambda)$ is the overlap integral, which defines the extent of overlap between the emission spectrum of the donor ($F_D(\lambda)$) and the absorption spectrum of the acceptor ($\varepsilon_A(\lambda)$, see Figure S2 for the graphical representation of this overlap. The value $J(\lambda)$ was calculated according the Equation (S3) [1]:

$$J(\lambda) = \int_0^\infty F_{\mathrm{D}}(\lambda)\varepsilon_{\mathrm{A}}(\lambda)\lambda^4 d\lambda \Big/ \int_0^\infty F_{\mathrm{D}}(\lambda) d\lambda \quad \text{(S3)}$$

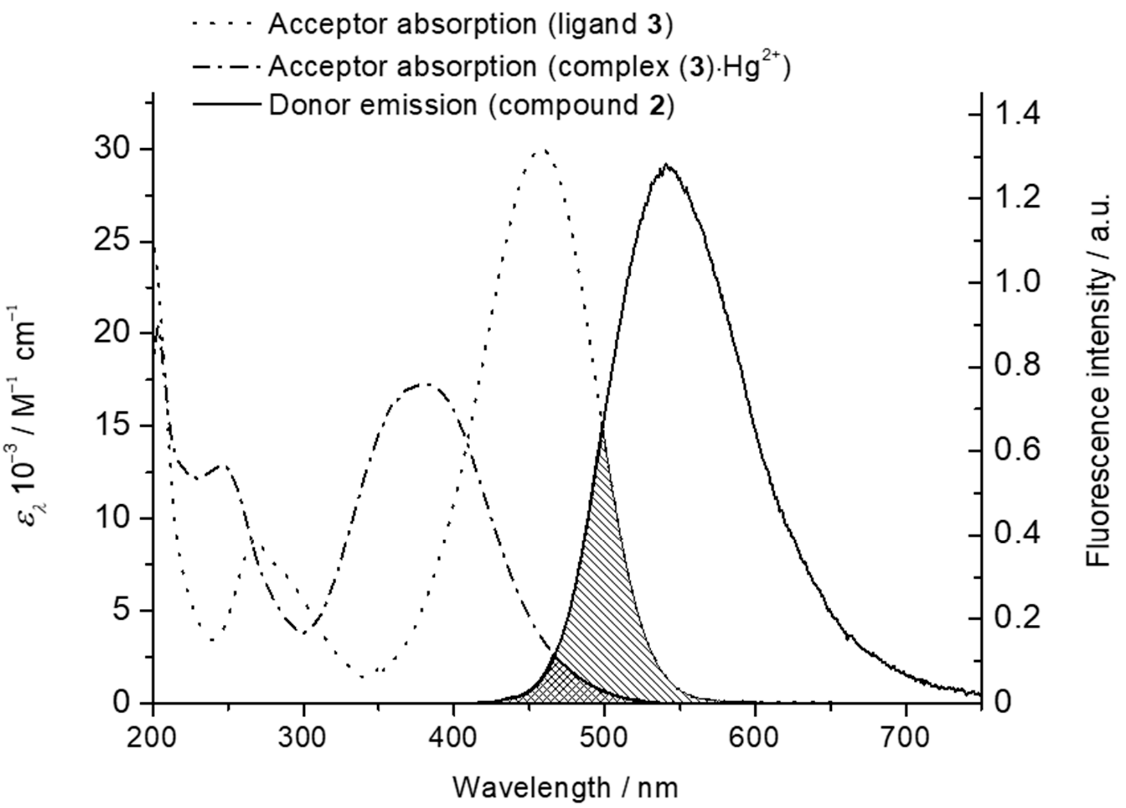

Figure S2. Overlap between the fluorescence emission spectrum of compound **2** and absorption spectra of **3** and (**3**)·Hg^{2+}. Concentration of all species 2 × 10^{-5} M. Excitation wavelength 380 nm.

Taking into account that κ^2 = 2/3 (for a random orientation) and $\varphi_{D,0}^{\mathrm{fl}}$ = 0.029 (the quantum yield of compound **2**, see Table 1 in the manuscript text), we found the RET efficiency Φ_{RET} to be as high as 0.91 for the free ligand **1** and 0.31 for the complex (**1**)·Hg^{2+}. Calculation results are summarized in Table S1.

Table S1. Various parameters of RET process occurring between energy donor (compound **2**) and energy acceptor (**3** and (**3**)·Hg^{2+}) chromophores

Donor–Acceptor pair	$J(\lambda)$ / $M^{-1}\cdot cm^{-1}\cdot nm^4$	R_0 / Å	r / Å	$\varphi_{D,0}^{\mathrm{fl}}$	Φ_{RET}
**2** (Donor)–**3** (Acceptor)	$2.52\cdot10^{14}$	22.7	15.3	0.029	0.91
**2** (Donor)–(**3**)·Hg^{2+} (Acceptor)	$1.06\cdot10^{13}$	13.4	15.3	0.029	0.31

2. Calculated Absorption and Emission Spectra of Ligands 1 and 3 and Complexes (1)·Hg^{2+} and (3)·Hg^{2+}

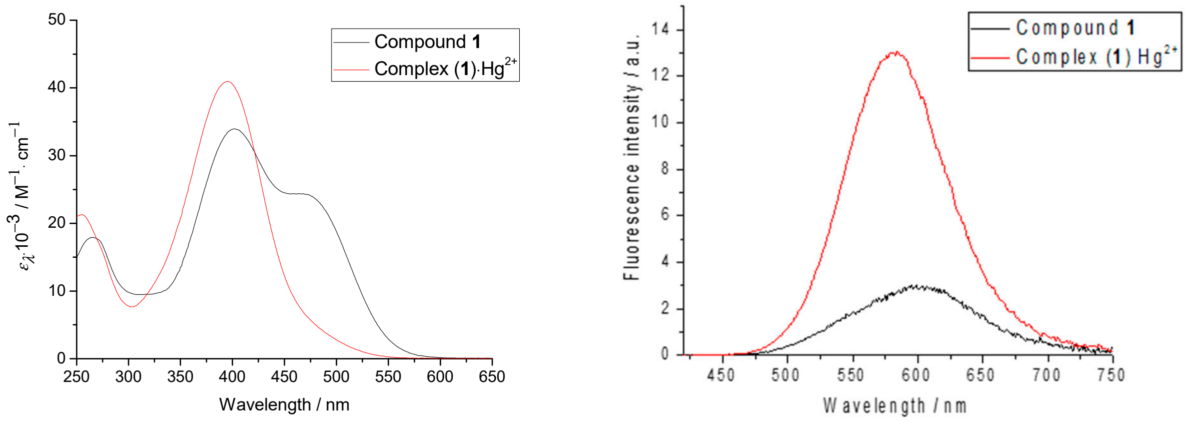

(**a**) (**b**)

Figure S3. Absorption (**a**) and fluorescence (**b**) spectra of the free ligand **1** and complex (**1**)·Hg^{2+} in water at pH 6.0 (acetate buffer, 0.01 M) calculated by SPECFIT/32 program.

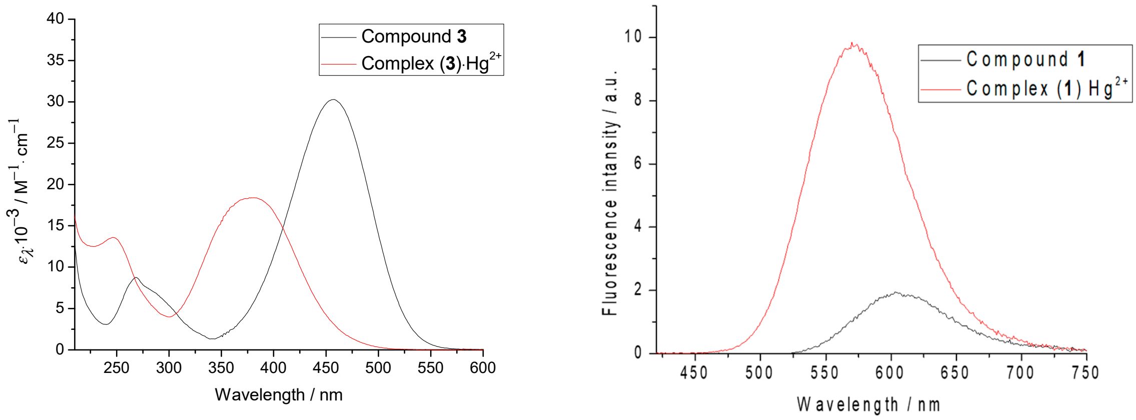

(**a**) (**b**)

Figure S4. Absorption (**a**) and fluorescence (**b**) spectra of the free ligand **3** and complex (**3**)·Hg^{2+} in water at pH 6.0 (acetate buffer, 0.01 M) calculated by SPECFIT/32 program.

3. Influence of Metal Cations on the Absorption and Emission Spectra of Compound 1

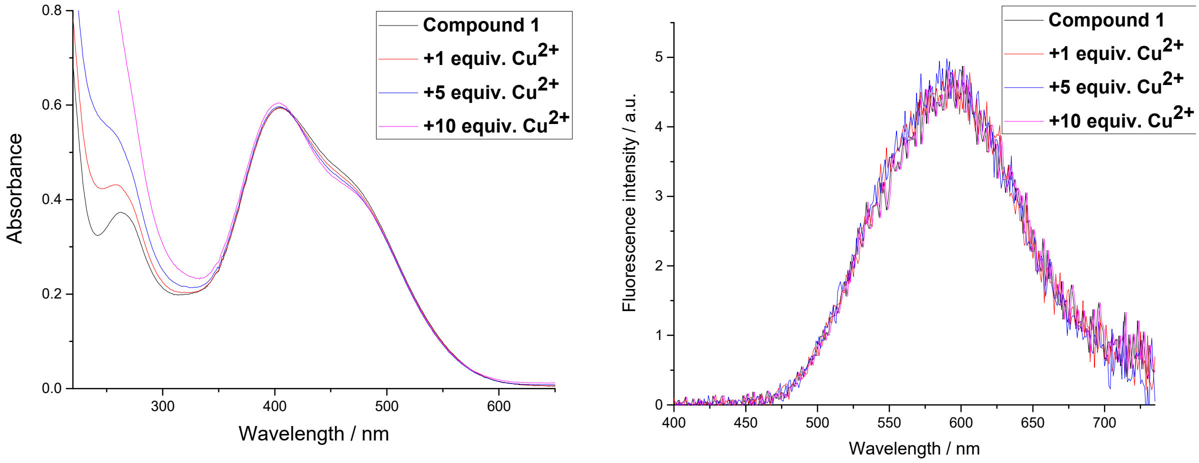

(**a**) (**b**)

Figure S5. Absorption (**a**) and fluorescence (**b**) spectra of compound **1** (2×10^{-5} M) in the absence and presence of 1, 5, and 10 equiv. of $Cu(ClO_4)_2$ in water at pH 6.0 (acetate buffer, 0.01 M). Excitation wavelength 380 nm.

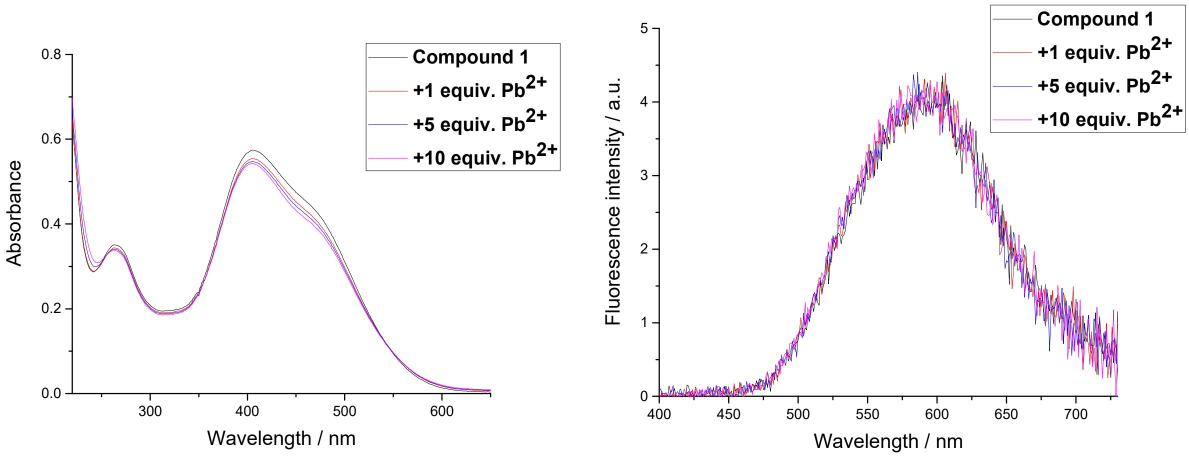

(**a**) (**b**)

Figure S6. Absorption (**a**) and fluorescence (**b**) spectra of compound **1** (2×10^{-5} M) in the absence and presence of 1, 5, and 10 equiv. of $Pb(ClO_4)_2$ in water at pH 6.0 (acetate buffer, 0.01 M). Excitation wavelength 380 nm.

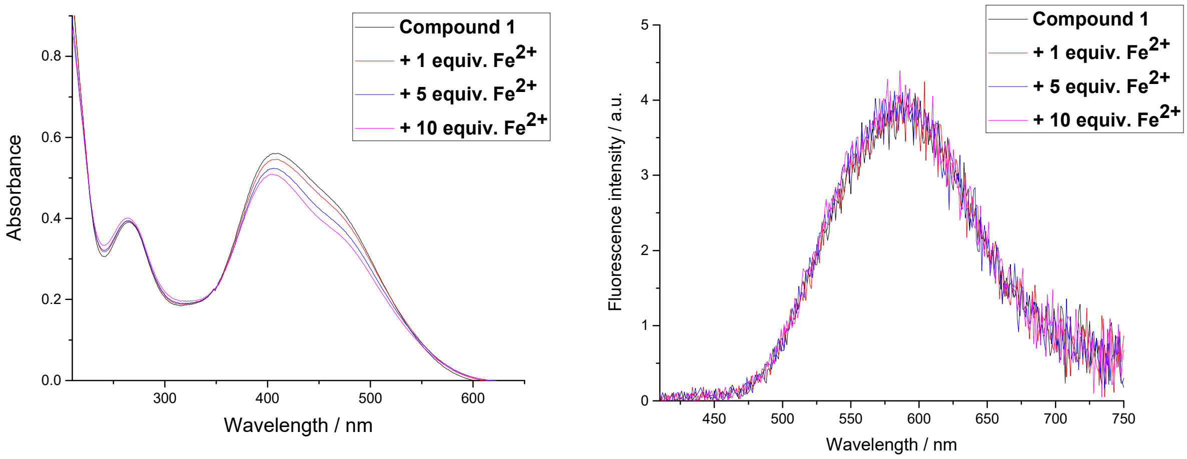

(**a**) (**b**)

Figure S7. Absorption (**a**) and fluorescence (**b**) spectra of compound **1** (2×10^{-5} M) in the absence and presence of 1, 5, and 10 equiv. of $Fe(ClO_4)_2$ in water at pH 6.0 (acetate buffer, 0.01 M). Excitation wavelength 380 nm

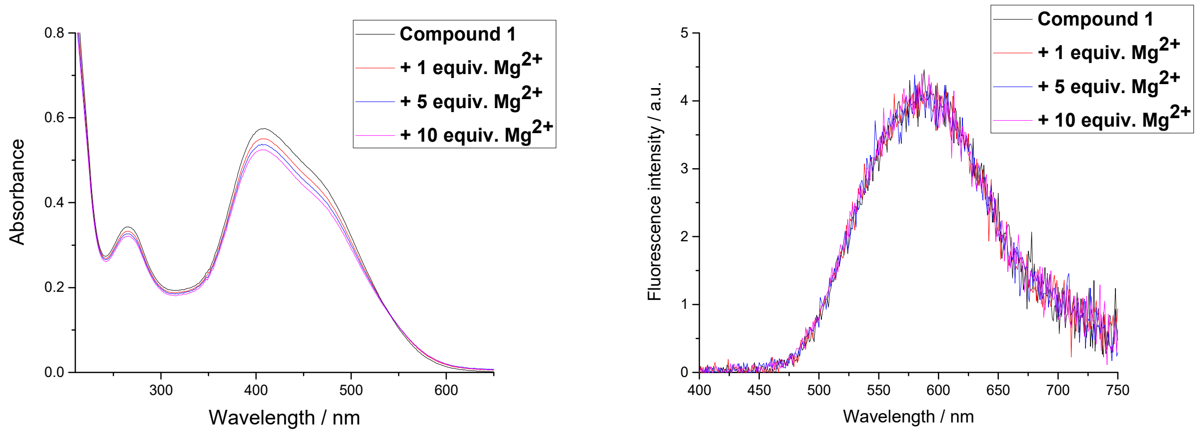

(a) (b)

Figure S8. Absorption (**a**) and fluorescence (**b**) spectra of compound **1** (2×10^{-5} M) in the absence and presence of 1, 5, and 10 equiv. of $Mg(ClO_4)_2$ in water at pH 6.0 (acetate buffer, 0.01 M). Excitation wavelength 380 nm

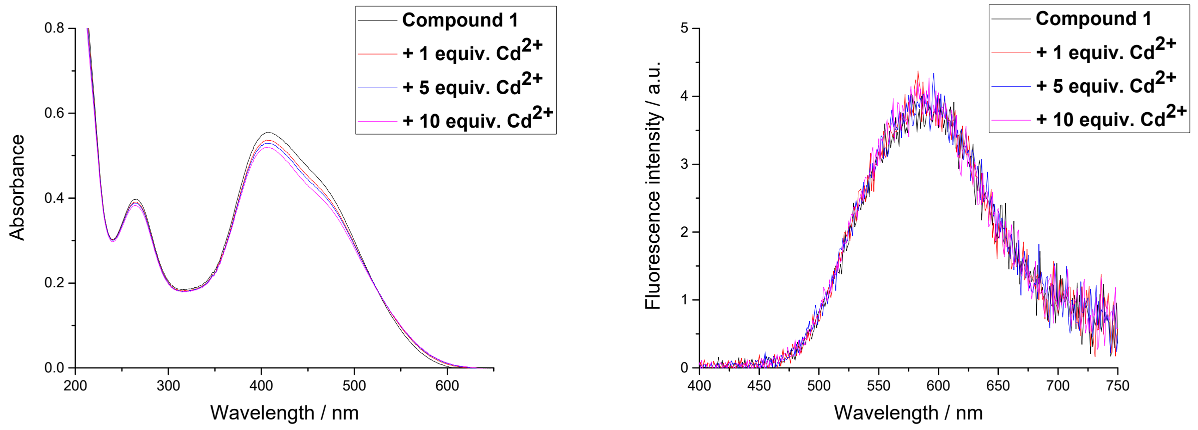

(a) (b)

Figure S9. Absorption (**a**) and fluorescence (**b**) spectra of compound **1** (2×10^{-5} M) in the absence and presence of 1, 5, and 10 equiv. of $Cd(ClO_4)_2$ in water at pH 6.0 (acetate buffer, 0.01 M). Excitation wavelength 380 nm

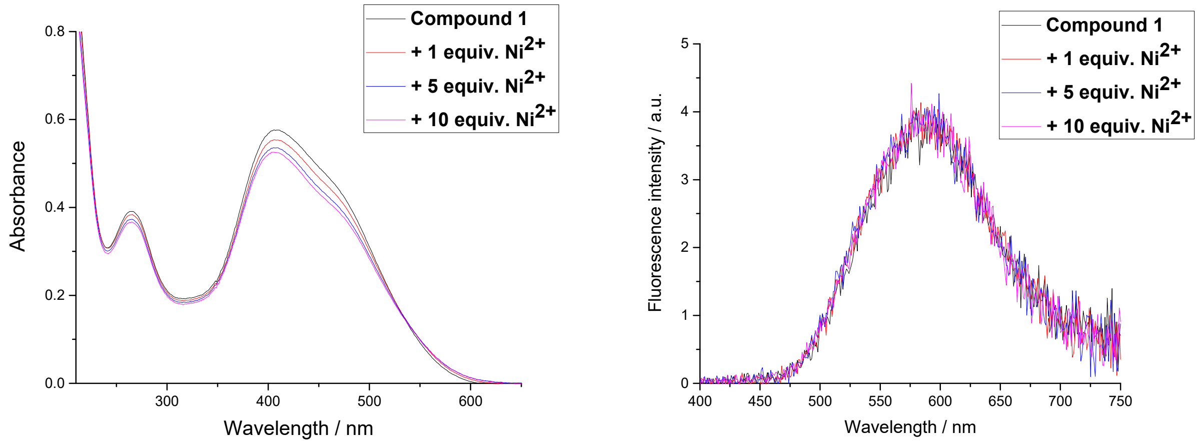

(a) (b)

Figure S10. Absorption (**a**) and fluorescence (**b**) spectra of compound **1** (2×10^{-5} M) in the absence and presence of 1, 5, and 10 equiv. of $Ni(ClO_4)_2$ in water at pH 6.0 (acetate buffer, 0.01 M). Excitation wavelength 380 nm.

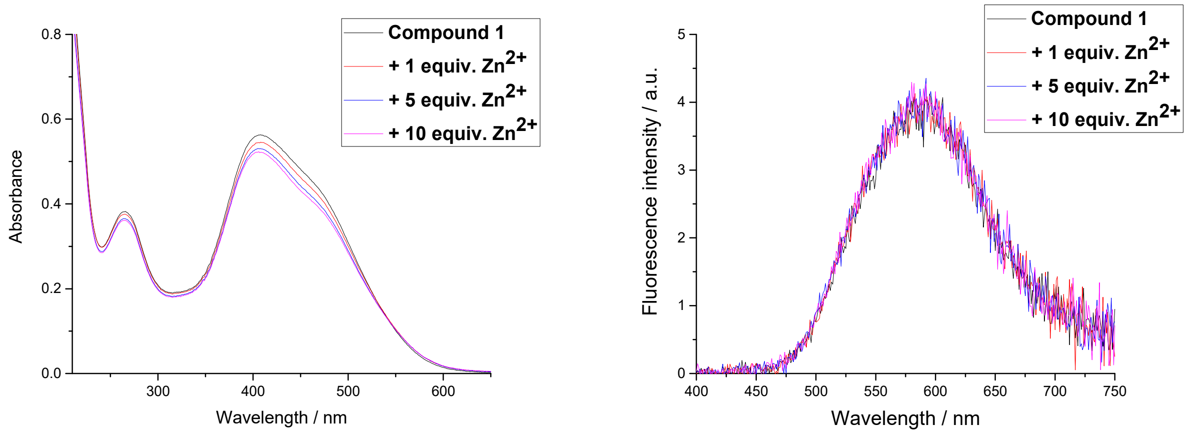

(**a**) (**b**)

Figure S11. Absorption (**a**) and fluorescence (**b**) spectra of compound **1** (2×10^{-5} M) in the absence and presence of 1, 5, and 10 equiv. of $Zn(ClO_4)_2$ in water at pH 6.0 (acetate buffer, 0.01 M). Excitation wavelength 380 nm.

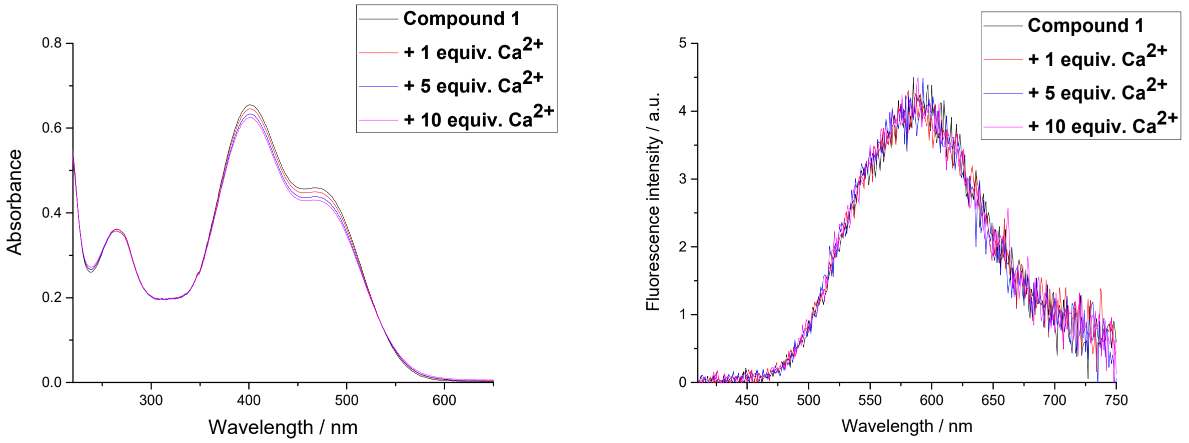

(**a**) (**b**)

Figure S12. Absorption (**a**) and fluorescence (**b**) spectra of compound **1** (2×10^{-5} M) in the absence and presence of 1, 5, and 10 equiv. of $Ca(ClO_4)_2$ in water at pH 6.0 (acetate buffer, 0.01 M). Excitation wavelength 380 nm.

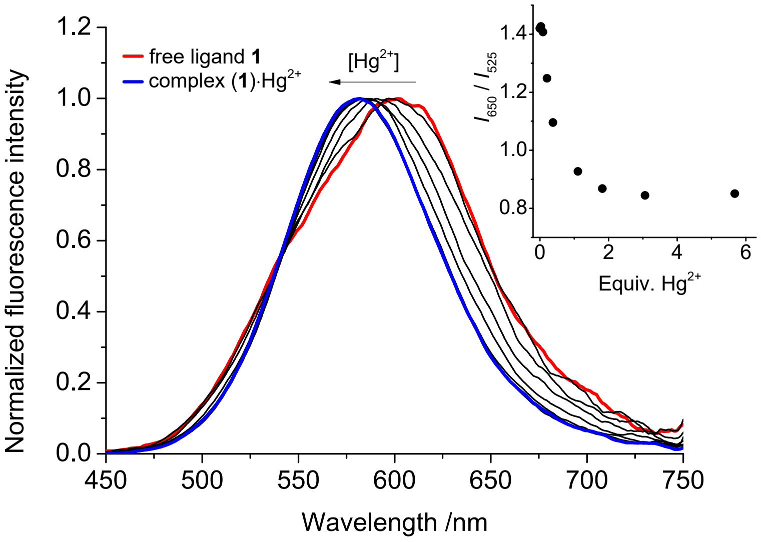

Figure S13. Changes in the normalized emission spectrum of compound **1** (20 μM) upon addition of mercury (II) perchlorate in water at pH 6.0 (acetate buffer solution, 0.01 M). Excitation wavelength was 380 nm. The upper insert shows the plot of the ratio of fluorescence intensities at 650 nm (I_{650}) and 525 nm (I_{525}) versus equivalents of Hg^{2+}.

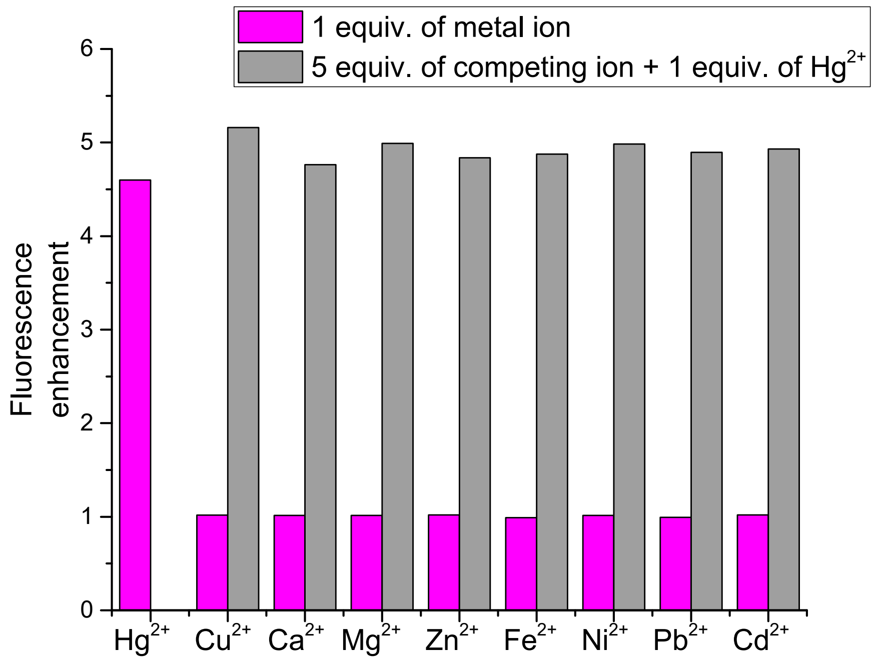

Figure S14. Selectivity of probe **1** (2×10^{-5} M) for Hg^{2+} in water at pH 6.0 (acetate buffer, 0.01 M). Excitation wavelength was 380 nm. Fluorescence enhancement value was calculated as the ratio of the emission intensity at 580 nm measured after the addition of metal ion to that measured before addition.

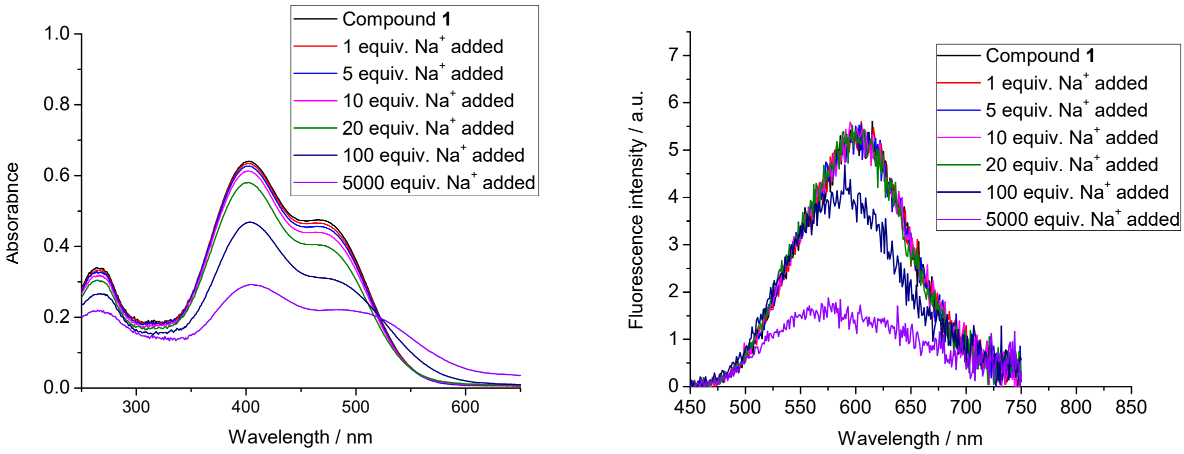

(**a**) (**b**)

Figure S15. Absorption (**a**) and fluorescence (**b**) spectra of compound **1** (2×10^{-5} M) in the absence and presence of 1, 5, 10, 20, 100, and 5000 equiv. of $NaClO_4$ in water. Excitation wavelength 380 nm.

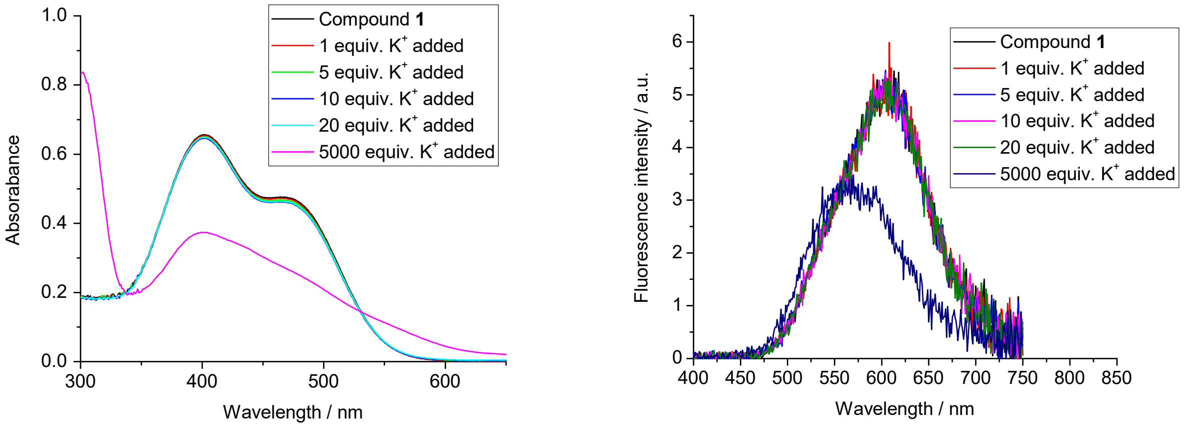

(**a**) (**b**)

Figure S16. Absorption (**a**) and fluorescence (**b**) spectra of compound **1** (2×10^{-5} M) in the absence and presence of 1, 5, 10, 20, and 5000 equiv. of KNO_3 in water. Excitation wavelength 380 nm**.**

4. Spectrophotometric and Spectrofluorometric Titration of Ligands 1, 2, and 3 with Hg^{2+}

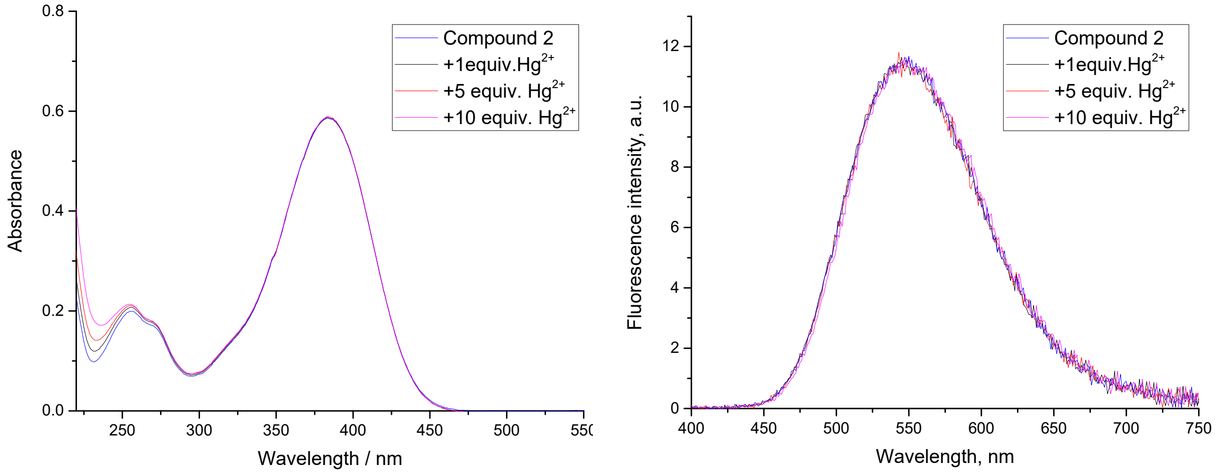

(**a**) (**b**)

Figure S17. Absorption (**a**) and fluorescence (**b**) spectra of compound **2** (2×10^{-5} M) in the absence and presence of 1, 5, and 10 equiv. of $Hg(ClO_4)_2$ in water at pH 6.0 (acetate buffer, 0.01 M). Excitation wavelength 380 nm.

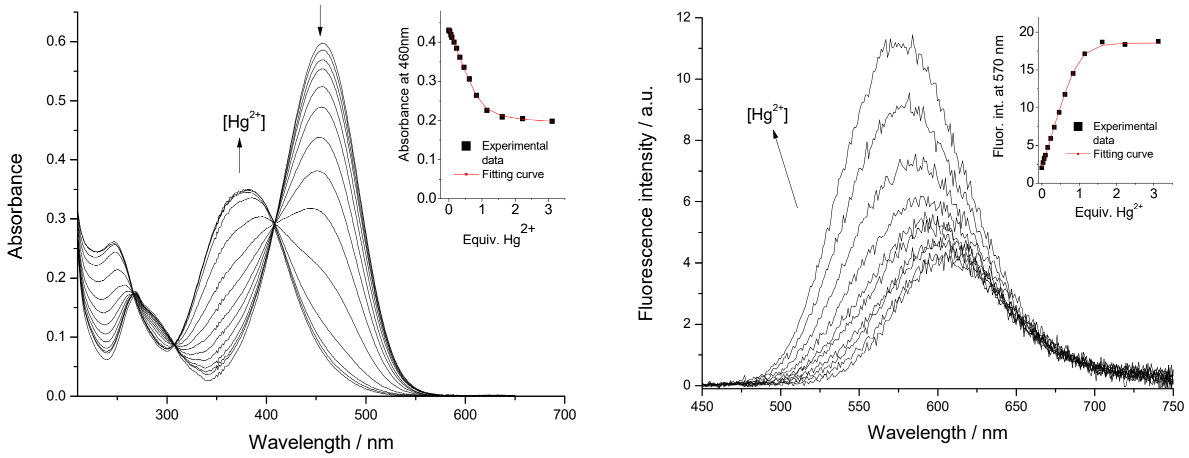

(**a**) (**b**)

Figure S18. Changes in the absorption (**a**) and fluorescence (**b**) spectra of compound **3** (2×10^{-5} M) upon addition of aliquots of $Hg(ClO_4)_2$ in water at pH 6.0 (acetate buffer, 0.01 M). Excitation wavelength 405 nm. The upper inserts show the absorbance at 460 nm (**a**) or fluorescence intensity at 570 nm (**b**) versus equivalents of Hg^{2+} added.

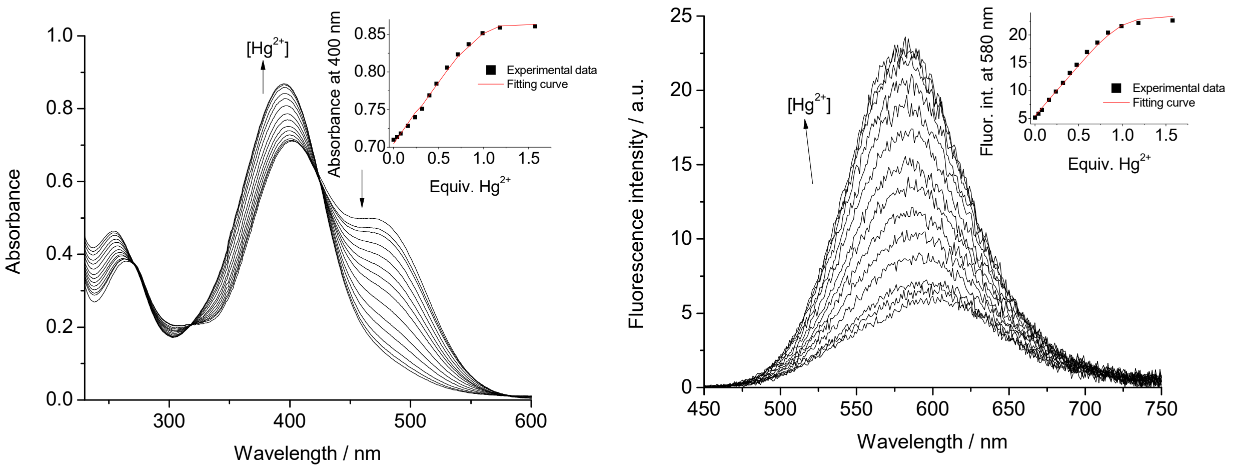

(**a**) (**b**)

Figure S19. Changes in the absorption (**a**) and fluorescence (**b**) spectra of compound **1** (2×10^{-5} M) upon addition of aliquots of $Hg(ClO_4)_2$ in water at pH 4.5 (acetate buffer, 0.01 M). Excitation wavelength 380 nm. The upper inserts show the absorbance at 400 nm (**a**) or fluorescence intensity at 580 nm (**b**) versus equivalents of Hg^{2+} added.

5. Cell Viability Studies

*5.1. Short Incubation Times of Cells with Compound **1** and $Hg(ClO_4)_2$*

In order to test if incubation of cells with $Hg(ClO_4)_2$ (2–50 μM for 15 min) and compound **1** (10 μM for 20 min) induced death of A549 cells (the main regime of cell treatment used in our work), the following live/dead cell assay was performed. A549 cells were seeded on round cover glasses placed in 24-well plates and grown for 24 h. Sowing density was 2×10^5 cells per ml. The cells were pre-incubated (15 min) with $Hg(ClO_4)_2$ at 2–50 μM, washed twice with Hanks' solution, incubated with **1** (10 μM, for 20 min) and washed twice with Hanks' solution. The fluorescent dyes Hoechst 33342 (10 μM) and propidium iodide (PI, 5 μM) were added to cells for 15 min, and the cells were examined under the inverted fluorescence microscope with a 40× objective (Axio Observer, Carl Zeiss AG, Oberkochen, Germany). Hoechst 33342 stains nuclei of all cells (living and dead ones), while PI stains nuclei of dead cells. A filter unit with the 530–585 nm excitation filter, the 600 nm dichroic mirror, and the 615 nm barrier emission filter was used to register fluorescent images of dead cells having red PI fluorescence in the nucleus. A filter unit with the 359–371 nm excitation filter, the 390 nm dichroic mirror, and the 410 nm barrier emission filter was used to register fluorescent images of all cells due to blue nuclear fluorescence of Hoechst33342. Both types of images were captured from each examined field of view, and total number of cells and dead cells were counted. At least 250–300 cells were examined in each sample.

It was found that after incubation of cells with compound **1** and 50 μM $Hg(ClO_4)_2$ the fraction of dead cells achieved 15–17%. When concentration of $Hg(ClO_4)_2$ reduced to 20 μM, the fraction of dead cells did not exceed 5–7% (Figure S20). In the control cells (without addition of any compounds), there were 2–3% of dead cells. The dead cells were morphologically different from the living ones that allowed us to recognize them in the transmitted-light images and exclude from the measurements.

*5.2. Long Incubation Time of Cells with Compound **1** and $Hg(ClO_4)_2$*

For the survival assays, A549 cells were seeded into 96-well plates. Sowing density was 2×10^4 cells per well. Twenty-four hours later, $Hg(ClO_4)_2$ (0.08–20 μM) or compound **1** (0.2–50 μM) was added gradually into the wells with a two-fold increment. The cytotoxicity was determined after 24 h incubation of cells with $Hg(ClO_4)_2$ or compound **1** by MTT assay. Compound **1** was added from the 100 mM stock solution in DMSO, and the final concentration of DMSO did not exceed 0.05%, which is non-toxic for cells even at long incubation time. MTT reagent (0.5 mg/ml) was added to cells 3 h before the end of incubation. At the end of incubation supernatant was removed, and 130 μl of DMSO was added to each well for 30 min

with gentle shaking (200 rpm) to dissolve the formed formazan crystals. Optical density readings were performed with the Uniplan photometer (Picon, Moscow, Russia) at the 492 nm wavelength.

The survival of cells exposed to $Hg(ClO_4)_2$ was calculated using the equation (S4):

$$\text{Survival} = \frac{A - A_{DMSO}}{A_0 - A_{DMSO}} \times 100\%\,, \tag{S4}$$

where A is the optical density value in the analyzed well, A_{DMSO} is the optical density in the control wells with DMSO only, and A_0 is the optical density in the control wells with cells without $Hg(ClO_4)_2$ or compound **1**. The survival of cells exposed to compound **1** was calculated using the equation (S5):

$$\text{Survival} = \frac{A - A_{DMSO} - A_1}{A_0 - A_{DMSO}} \times 100\%\,, \tag{S5}$$

where A_1 is the optical density value in the control wells with compound **1** (the same concentration as in the analyzed well) at 492 nm. All the measurements were performed in triplicates, and the results were averaged.

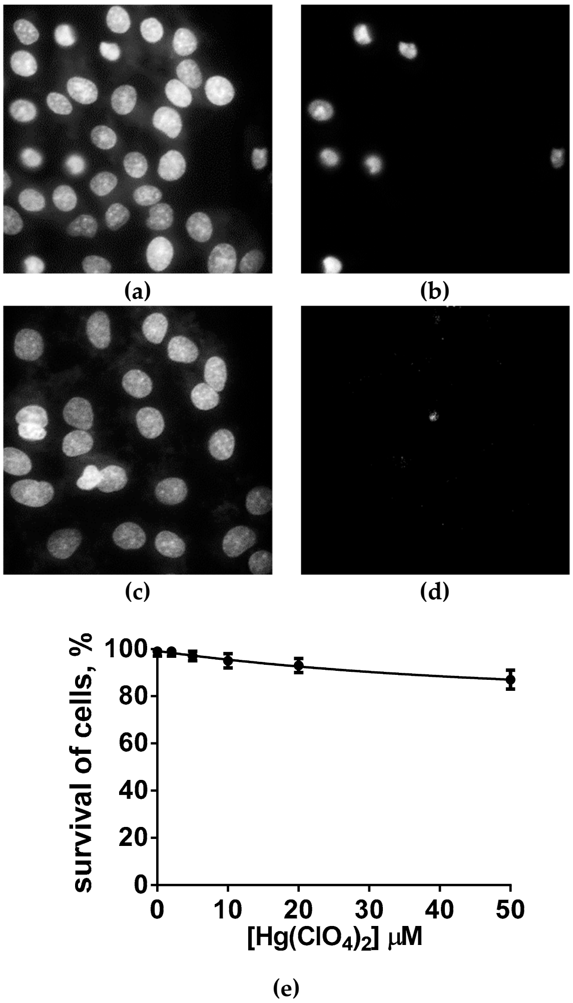

Figure S20. Survival of A549 cells incubated with $Hg(ClO_4)_2$ (2–50 µM, for 15 min) and with **1** (10 µM, for 20 min). (**a**–**d**) The fluorescence images of all cells stained with Hoechst 33342 (**a**, **c**) and dead cells stained with PI (**b**, **d**). Cells were incubated with 1 and 50 µM $Hg(ClO_4)_2$ (**a**, **b**) or 20 µM $Hg(ClO_4)_2$ (**c**, **d**). Bar represents 10 µm. (**e**) Survival of A549 cells at different concentrations of $Hg(ClO_4)_2$ in the presence of compound 1 (10 µM).

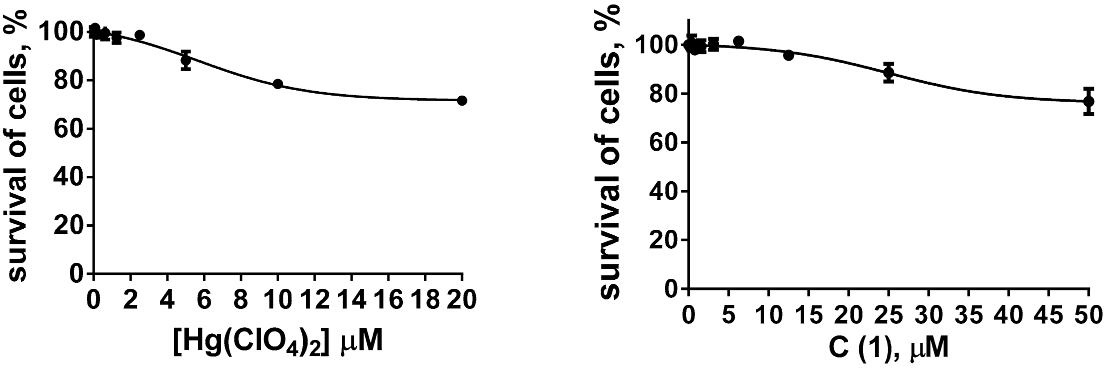

(**a**) (**b**)

Figure S21. Survival of A549 cells at different concentrations of $Hg(ClO_4)_2$ (**a**) or compound **1** (**b**) according to MTT assay. The cells were incubated with $Hg(ClO_4)_2$ or compound **1** for 24 h.

Growth of cells was affected by $Hg(ClO_4)_2$ at concentrations higher than 5 μM. The effect achieved *ca*. 30% at the $Hg(ClO_4)_2$ concentration of 20 μM (Figure S21a). Growth of cells was affected by compound **1** at concentration of 25 μM and higher. The effect achieved *ca*. 20% at the 50 μM concentration of compound **1** (Figure S21b).

6. Intracellular Localization of Compounds 3 and 1

To confirm accumulation of **3** in mitochondria, cells were incubated with 1 μM rhodamine 123 (Rh123; 15 min, 37 °C) after incubation with **3** (5 μM for 10 min, 37 °C) and studied with CLSM (Figure S22). Fluorescence of **3** and Rh123 was excited with λ_{ex} = 488 nm, and emission was registered within the 500–540 and 600–700 nm ranges. Control cells were separately incubated with **3** or Rh123 and measured at the same conditions. Fluorescence spectra of **3** and Rh123 overlap considerably, and thereby an "unmixing" procedure was applied to correct confocal images for fluorophore signal crosstalks according to equations (S6) and (S7):

$$I_{\mathrm{Rh123}} = \frac{I_{500-540} - \beta \times I_{600-700}}{1 - \alpha \times \beta}, \quad \text{(S6)}$$

$$I_3 = \frac{I_{600-700} - \alpha \times I_{500-540}}{1 - \alpha \times \beta}, \quad \text{(S7)}$$

where I_{Rh123} and I_3 are "unmixed" fluorescence intensities of Rh123 and **3** in the 500–540 and 600–700 nm spectral ranges, respectively; $I_{500-540}$ and $I_{600-700}$ are measured fluorescence intensities in the 500–540 and 600–700 nm spectral ranges, respectively; α—crosstalk coefficient for Rh123 in the 600–700 nm spectral range (α = 0.27 ± 0.02); β – crosstalk coefficient for **3** in the 500–540 nm spectral range (β = 0.40 ± 0.03).

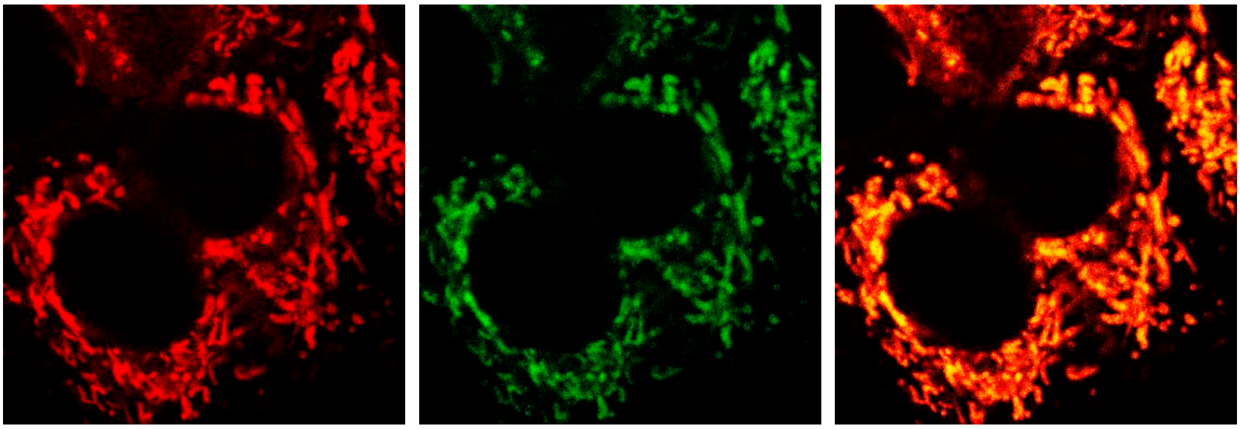

(**a**) (**b**) (**c**)

Figure S22. Typical CLSM images of A549 cells stained with **3** and rhodamine 123, a fluorescent probe of mitochondria. (**a**, **b**) Intracellular distributions of 3 (**a**) and rhodamine 123 (**b**). Bar represents 10 μm. (**c**) Merged images (**a**) and (**b**). Yellow color indicates colocalization of **3** and rhodamine 123. Fluorescent images were corrected for fluorophore signal crosstalks (see text for details).

Comparing distribution of fluorescence of **1** with the distribution of lipid droplets, which can be observed in transmitted-light images, it was concluded that **1** does not accumulate in lipid droplets (Figure S23).

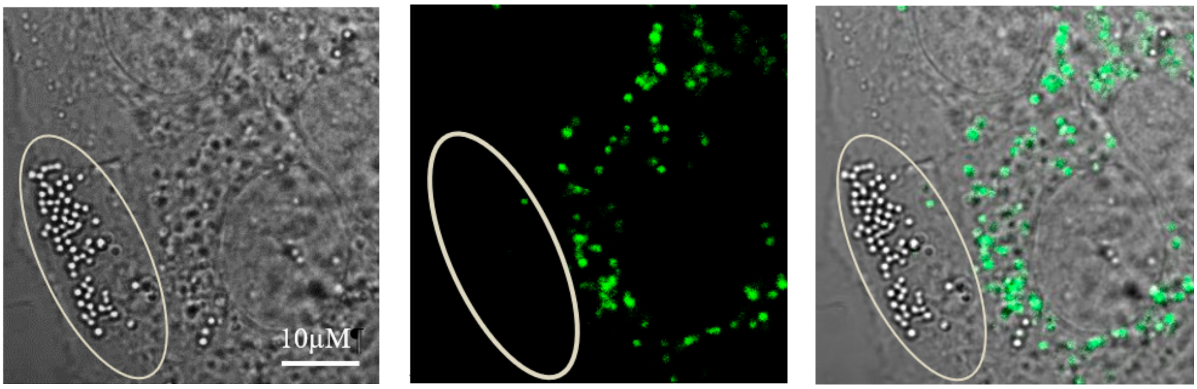

(**a**) (**b**) (**c**)

Figure S23. Accumulation sites of **1** in A549 cells are different from lipid droplets. (**a**) Transmitted light image of cells. An oval marks a group of lipid droplets situated at a cell periphery and seen as bright white granules. (**b**) A confocal fluorescent image showing intracellular distribution of compound **1**. (**c**) Merged images (**a**) and (**b**) demonstrate absence of **1** accumulation in lipid droplets.

We assumed that vesicular distribution of **1** in cells is related to its localization in lysosomes. To prove this assumption, it was necessary to use a vital fluorescent probe that accumulates in lysosomes, but fluorescence spectra of most available probes overlap considerably or totally with a very wide fluorescence spectrum of **1**. Finally, we have selected chlorin *e*6 derivative Chl-CBDC (Figure S24) that accumulates in lysosomes, fluoresces in the 630–680 nm, and can be selectively excited at 632 nm [3]. Cells were pre-incubated with Chl-CBDC (0.5 µM for 1.5 h) and further incubated with compound **1** (10 µM for 20 min). To measure intracellular distribution of **1** and Chl-CBDC, a consequent scanning mode was applied. During the first scan, fluorescence of **1** was excited at 488 nm and recorded in the 500–600 nm range. During the consequent scan, fluorescence of Chl-CBDC was excited selectively at 632 nm and recorded in the 650–700 nm range (Figure S25).

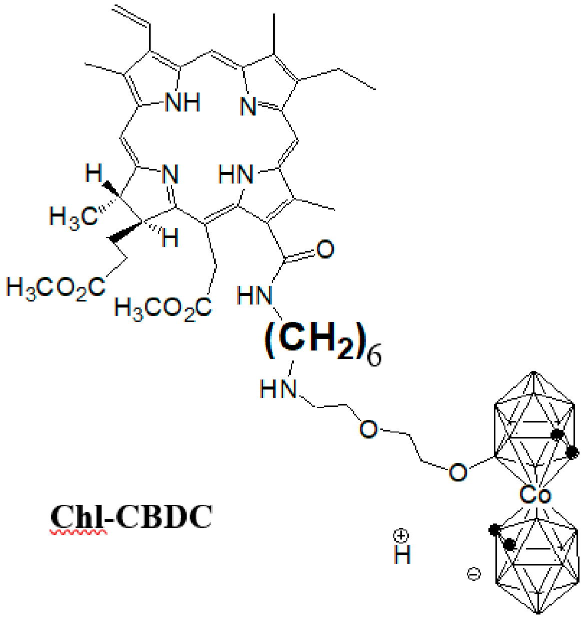

Chl-CBDC

Figure S24. Structure of chlorin *e*6 conjugate with cobalt bis(dicarbollide) nanocluster.

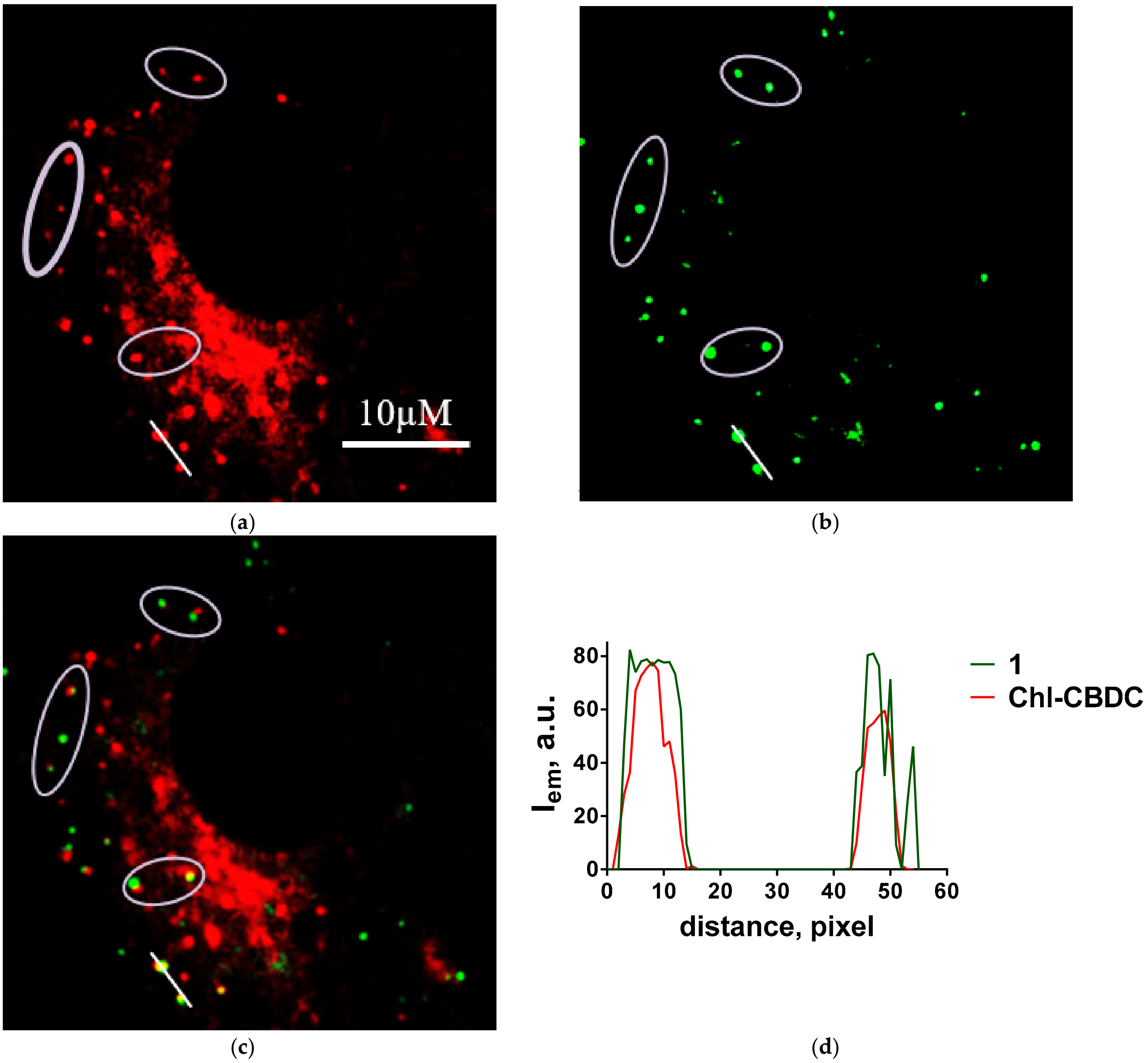

Figure S25. Identification of accumulation sites of **1** in A549 cells. (**a**) A confocal fluorescent image showing intracellular distribution of Chl-CBDC, which (as demonstrated previously [3]) accumulates in lysosomes. (**b**) A confocal fluorescent image showing intracellular distribution of compound **1**. (**c**) Merged images (**a**) and (**b**) demonstrate colocalization of **1** and Chl-CBDC in vesicles assigned to lysosomes on the basis of known intracellular localization of Chl-CBDC [3]. Ovals highlight regions, where colocalization of **1** and Chl-CBDC is most obvious. (**d**) Distribution of fluorescence intensities of **1** and Chl-CBDC along a white line passing through two vesicles (see panels (**a**)–(**c**)). Close similarity of both intensity profiles confirm colocalization of **1** and Chl-CBDC in these vesicles.

7. Intracellular Fluorescence Spectra of 1 in A549 Cells

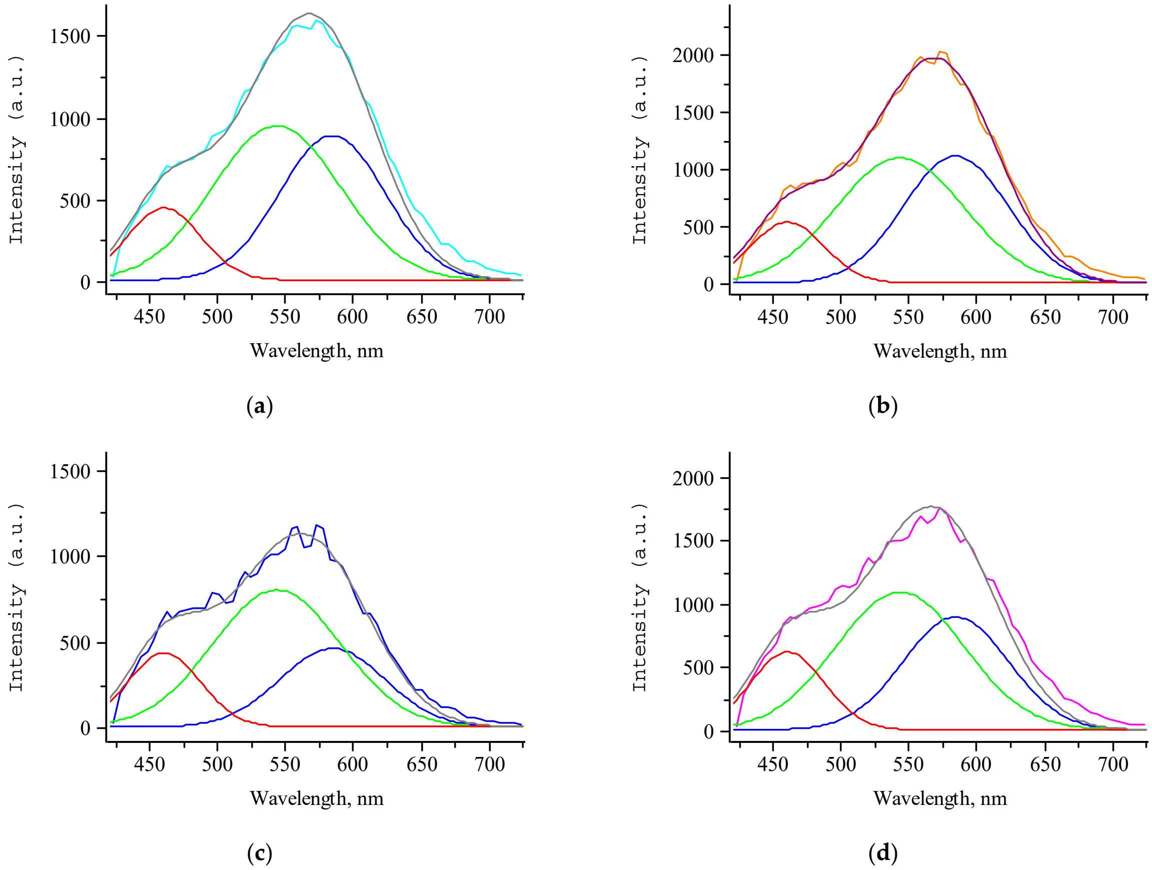

Figure S26. Examples of intracellular spectra of **1** (λ_{ex} = 405 nm**)** and their presentation as a superposition of fluorescence spectra of **2** (λ_{max} = 543 nm, green line), **3** (λ_{max} = 584 nm, blue line), and a residual fluorescence spectrum (λ_{max} = 459 nm, red line). Black line is calculated spectrum, which overlaps with the experimental spectrum. Panels (**a**)–(**d**) correspond to intracellular spectra in different parts of a cell.

8. Kinetics of Intracellular Accumulation and Retention of 1

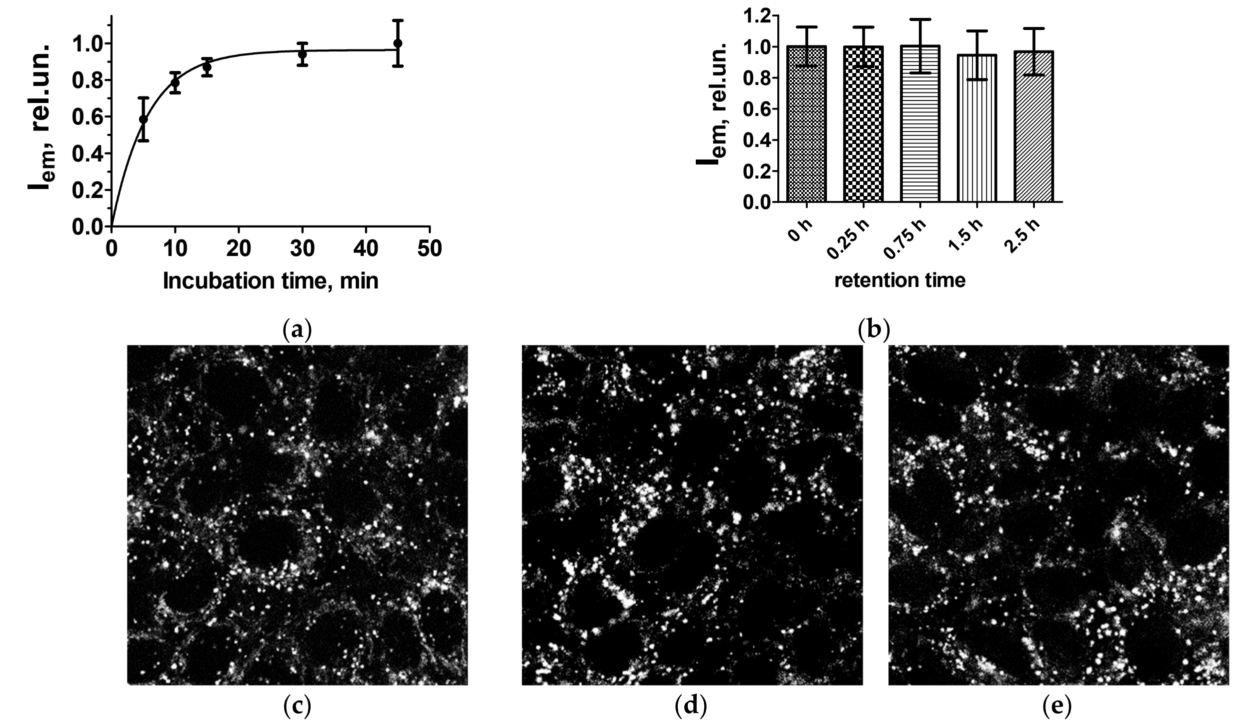

Figure S27. Kinetics of accumulation (**a**) and retention (**b**–**e**) of **1** (10 μM) in A549 cells. (**a**) Cells were incubated with **1** (10 μM) during different periods of time and recorded with CLSM at the identical parameters of measurements. Fluorescence intensity (I_{em}) of **1** in cells was averaged over 30–50 cells and presented as the mean value ±SEM for each incubation time. (**b**–**e**) Cells were incubated with **1** (10 μM) for 30 min, placed in the fresh medium (without **1**) for different (0–2.5 h) periods of time, and recorded with CLSM at the identical parameters of measurements. Fluorescence intensity (I_{em}) of **1** in cells was averaged over 30–50 cells and presented as the mean value ±SEM for each incubation time after removal of **1** from culture medium (**b**). (**c**–**e**) CLSM images of **1** in A549 cells at different periods of time after removal of **1** from culture medium: 0 h (**c**), 0.75 h (**d**), and 2.5 h (**e**). Bar corresponds to 30 μM. Compound **1** is characterized by long retention in cells.

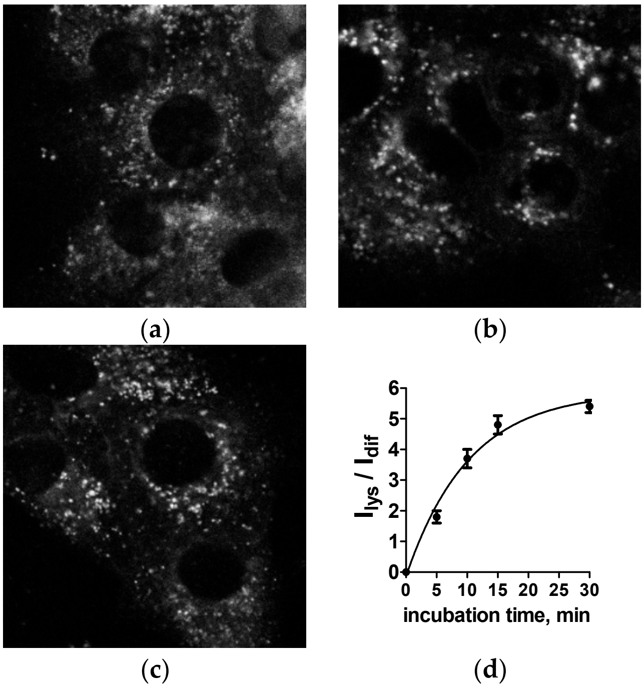

Figure S28. Intracellular distribution of compound **1** in 5 (**a**), 10 (**b**), and 30 (**c**) min incubation of A549 cells with dye **1** (20 μM) and the ratio of fluorescent intensities in lysosomes (I_{lys}) to diffuse component (I_{dif}), which increases as a function of incubation time (**d**). Fluorescence of **1** was recorded in the 500–600 nm spectral range using confocal microscopy. Bar represents 10 μm.

9. Probing Interactions of 1 and 3 with Hg^{2+} in Cells

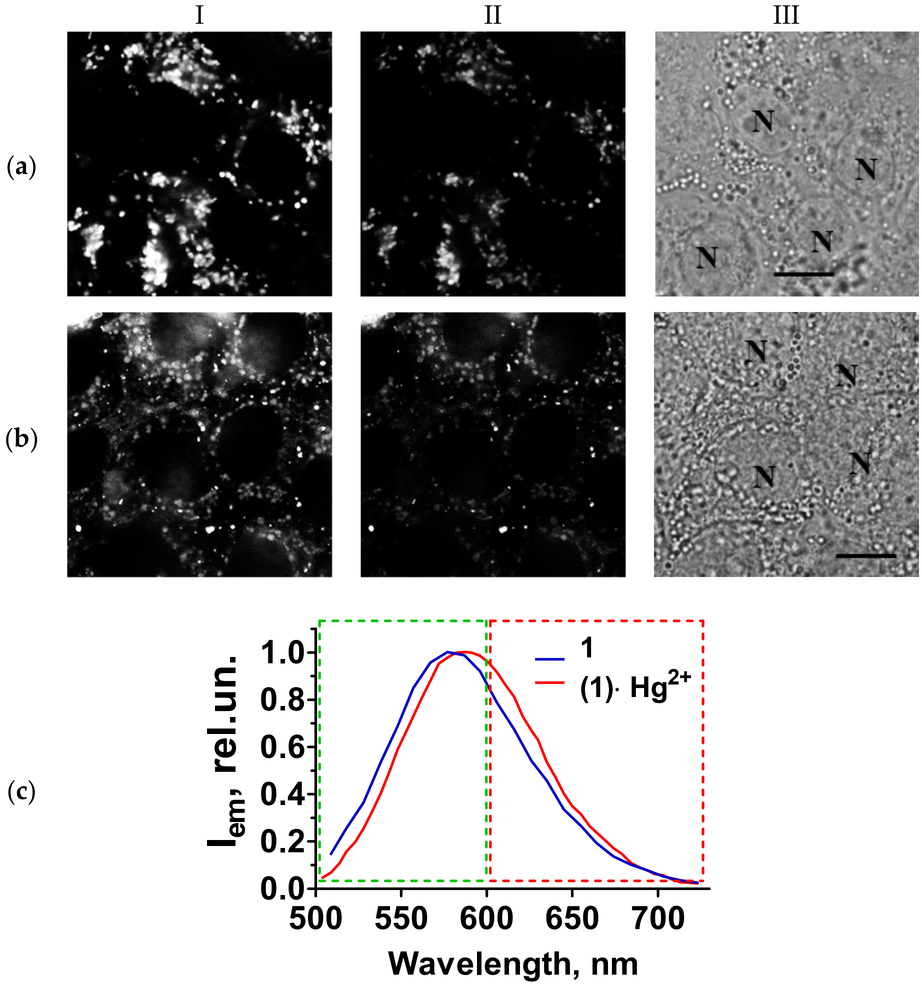

Figure S29. Confocal fluorescence images (**a, b**) and fluorescence spectra (**c**) of **1** and (**1**)·Hg^{2+} in A549 cells at λ_{ex} = 488 nm. (Row **a**) Cells were incubated with **1** (10 μM) for 20 min. (Row **b**) Cells were pre-incubated with 20 μM of $Hg(ClO_4)_2$ for 15 min, washed twice with Hanks' solution, and incubated with 10 μM of **1** for 20 min. Images of intracellular fluorescence were measured in the 500–600 nm (column I) and 600–730 nm (II column) spectral ranges. Transmitted light images of cells are shown in column III. Bar represents 10 μm. N marks a nucleus. (**c**) Typical normalized intracellular fluorescence spectra of **1** and (**1**)·Hg^{2+}. Green and red rectangles show two spectral regions (500–600 and 600–730 nm) selected for image recording. The ratio of the average fluorescence intensity in the green channel (500–600 nm) to the average fluorescence intensity in the red channel (600–730 nm) was R = 1.78 ± 0.04 in the absence of Hg^{2+} ions, and 1.46 ± 0.04 for the complex (**1**)·Hg^{2+}.

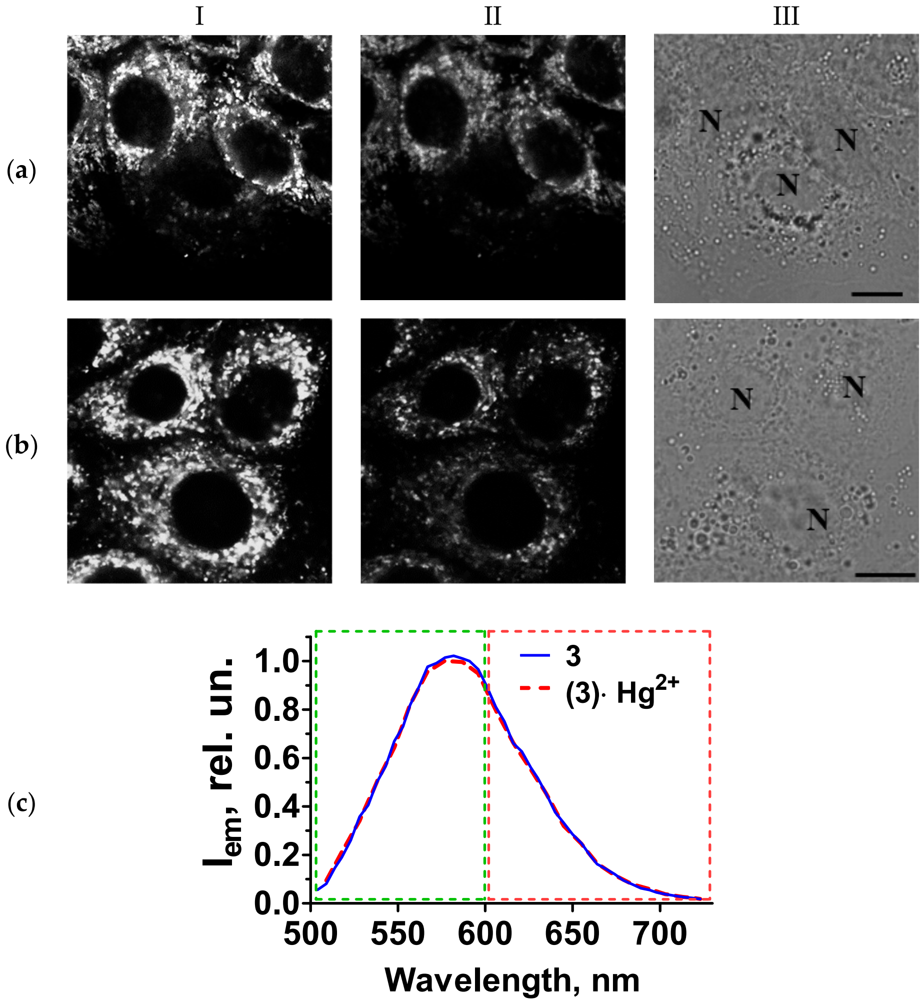

Figure S30. Confocal fluorescence images (**a**, **b**) and fluorescence spectra (**c**) of **3** and (**3**)·Hg^{2+} in A549 cells at λ_{ex} = 488 nm. (Row **a**) Cells were incubated with **3** (10 μM) for 20 min. (Row **b**) Cells were pre-incubated with 20 μM of $Hg(ClO_4)_2$ for 15 min, washed twice with Hanks' solution, and incubated with 10 μM of **3** for 20 min. Images of intracellular fluorescence were measured in the 500–600 nm (column I) and 600–730 nm (column II) spectral ranges. Transmitted light images of cells are shown in column III. Bar represents 10 μm. N marks nucleus. (**c**) Typical normalized intracellular fluorescence spectra of **3** and (**3**)·Hg^{2+}. Green and red rectangles show two spectral regions (500–600 and 600–730 nm) selected for image recording.

10. Interactions of 1 with Cu^{2+} and Pb^{2+} in Living Cells

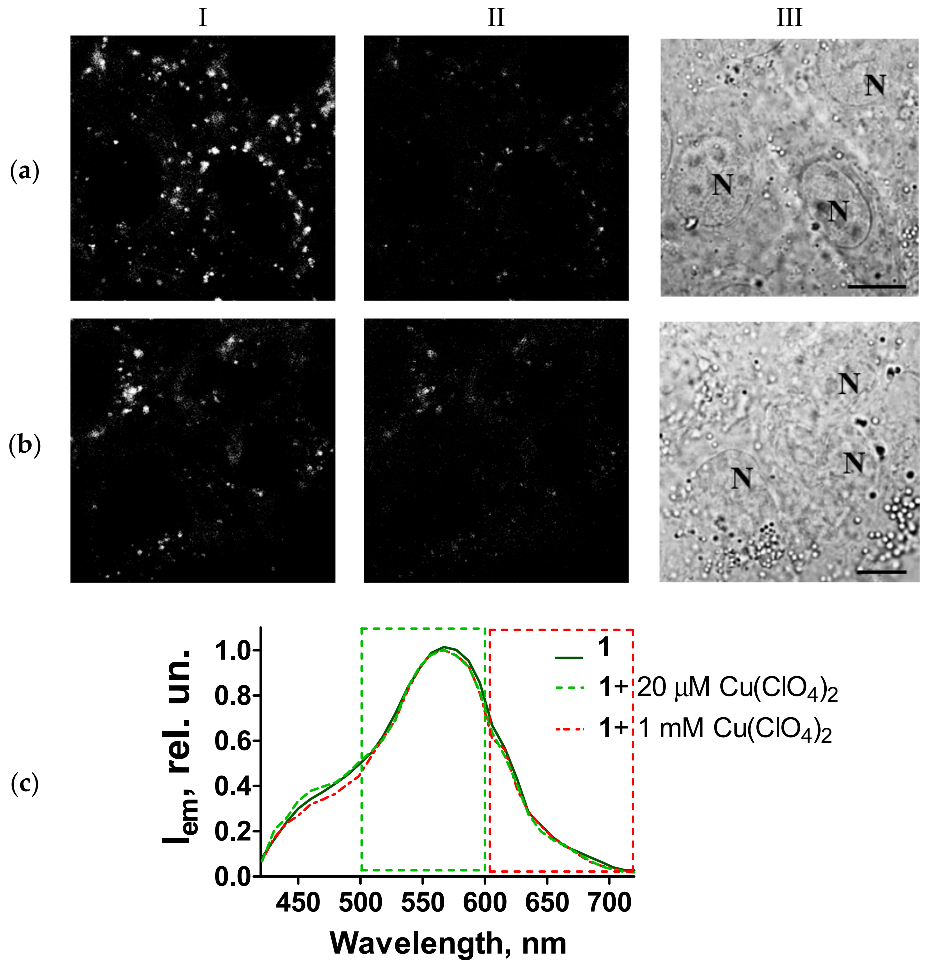

Figure S31. Confocal fluorescence images (**a, b**) and fluorescence spectra (**c**) of **1** (λ_{ex} = 405 nm) in A549 cells pre-incubated with $Cu(ClO_4)_2$. Cells were pre-incubated with $Cu(ClO_4)_2$ at 20 µM (**a**) or 1 mM (**b**) for 15 min, washed twice with Hanks' solution, and incubated with 10 µM of **1** for 20 min. Images of intracellular fluorescence were measured in the 500–600 nm (column I) and 600–730 nm (column II) spectral ranges. Transmitted light images of cells are shown in column III. Bar represents 10 µm. N marks a nucleus. (**c**) Typical normalized fluorescence spectra of **1** in cells non-treated and treated with $Cu(ClO_4)_2$. Green and red rectangles show two spectral regions (500–600 and 600–730 nm) selected for image recording. The ratio *R* of the average fluorescence intensity in the green channel (500–630 nm) to the average fluorescence intensity in the red channel (600–730 nm) was 4.4 ± 0.2 in the absence of $Cu(ClO_4)_2$, 4.1 ± 0.1 at 20 µM $Cu(ClO_4)_2$, and 4.3 ± 0.2 at 1 mM $Cu(ClO_4)_2$.

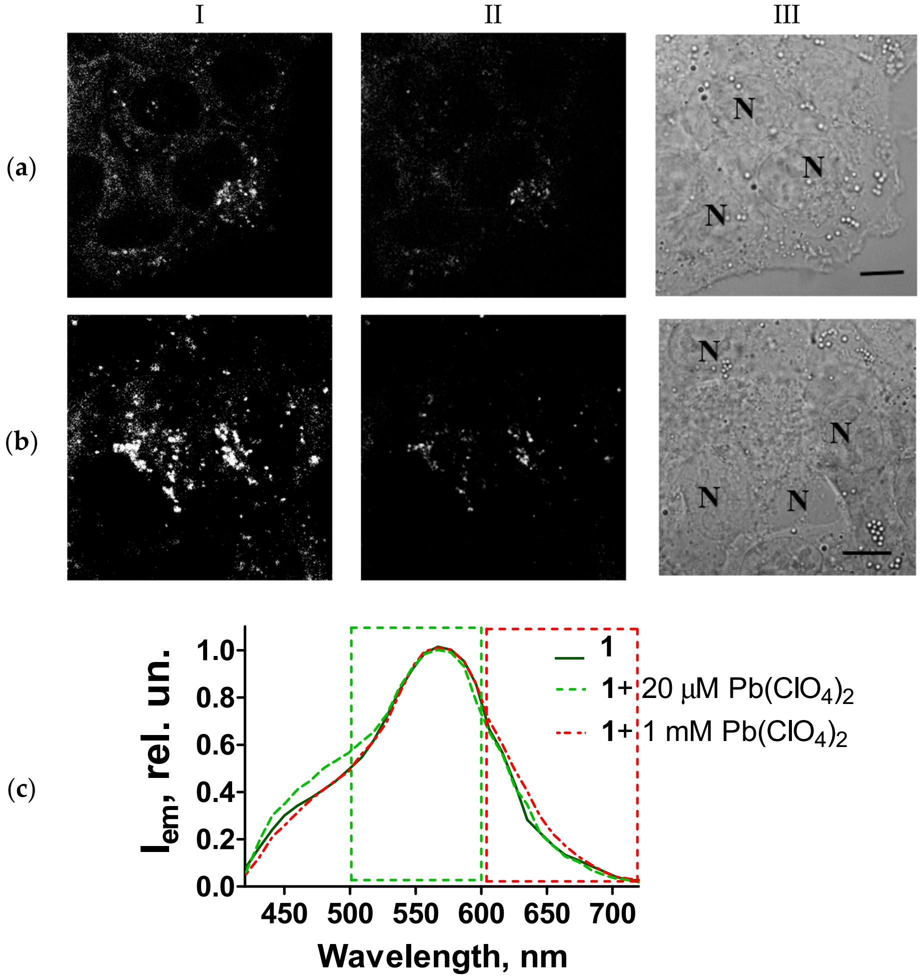

Figure S32. Confocal fluorescence images (**a, b**) and fluorescence spectra (**c**) of **1** (λ_{ex} = 405 nm) in A549 cells pre-incubated with $Pb(ClO_4)_2$. Cells were pre-incubated with $Pb(ClO_4)_2$ at 20 μM (**a**) or 1 mM (**b)** for 15 min, washed twice with Hanks' solution, and incubated with 10 μM of **1** for 20 min. Images of intracellular fluorescence were measured in the 500–600 nm (column I) and 600–730 nm (column II) spectral ranges. Transmitted light images of cells are shown in column III. Bar represents 10 μm. N marks a nucleus. (**c**) Typical normalized fluorescence spectra of **1** in cells non-treated and treated with $Pb(ClO_4)_2$. Green and red rectangles show two spectral regions (500–600 and 600–730 nm) selected for image recording. The ratio *R* of the average fluorescence intensity in the green channel (500–630 nm) to the average fluorescence intensity in the red channel (600–730 nm) was 4.4 ± 0.2 in the absence of $Pb(ClO_4)_2$, 4.2 ± 0.1 at 20 μM $Pb(ClO_4)_2$, and 4.2 ± 0.2 at 1 mM $Pb(ClO_4)_2$.

11. Alternative Choice of Spectral Ranges for Ratiometric Detection of Hg^{2+} in Cells

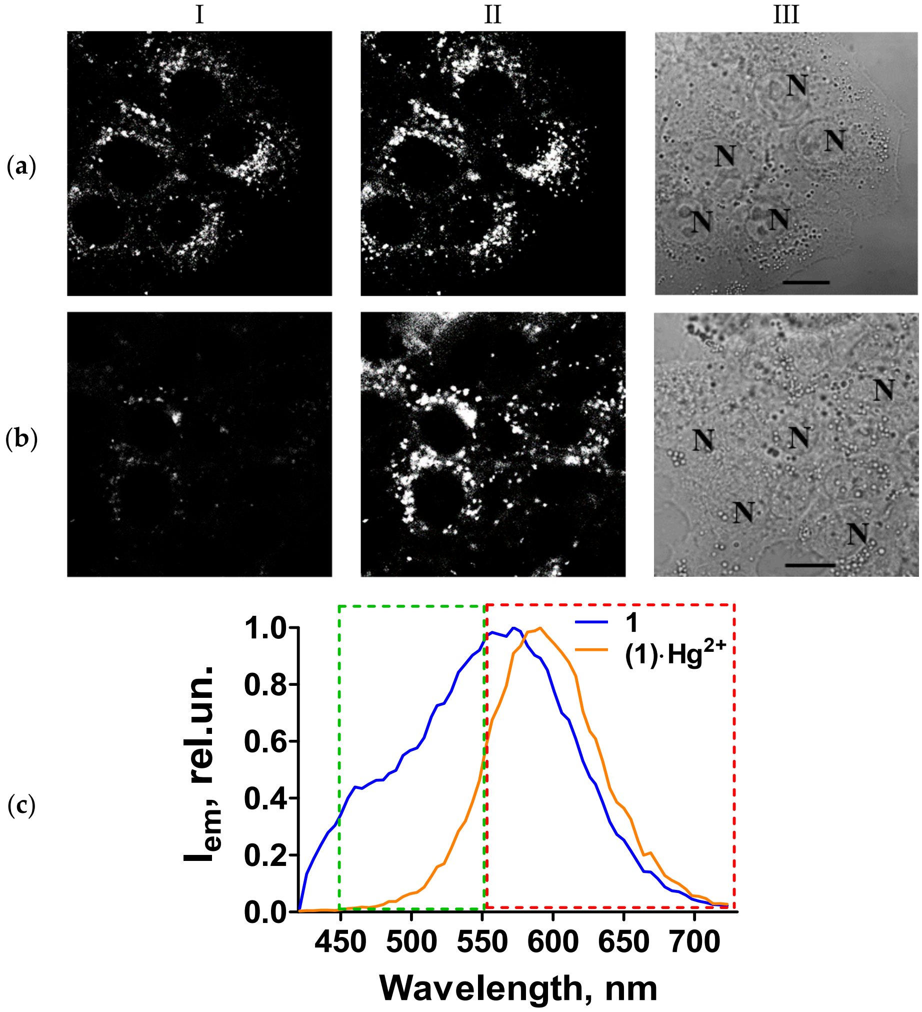

Figure S33. Confocal fluorescence images (**a, b**) and fluorescence spectra (**c, d**) of **1** and (1)·Hg^{2+} in A549 cells at λ_{ex} = 405 nm: alternative choice of spectral ranges for ratiometric measurements. (Row **a**) Cells were incubated with **1** (10 μM) for 20 min. (Row **b**) Cells were pre-incubated with 20 μM of $Hg(ClO_4)_2$ for 15 min, washed twice with Hanks' solution, and incubated with 10 μM of **1** for 20 min. Images of intracellular fluorescence were measured in the 450–550 nm (column I) and 550–730 nm (column II) spectral ranges at λ_{ex} = 405 nm. Transmitted light images of cells are shown in column III. Bar represents 10 μm. N marks a nucleus. (*c*) Typical normalized fluorescence spectra of **1** and (**1**)·Hg^{2+}. Green and red rectangles indicate two spectral regions (450–550 and 550–730 nm) selected for recording of images shown in columns I and II. The ratio of the average fluorescence intensity in the red channel (550–730 nm) to the average fluorescence intensity in the green channel (450–550 nm) was $R = 1.7 \pm 0.2$ in the absence of Hg^{2+} ions, and 4.8 ± 0.3 for the complex (**1**)·Hg^{2+}.

12. Influence of Cysteine Addition on the Absorption and Emission Spectrum of (1)·Hg^{2+}

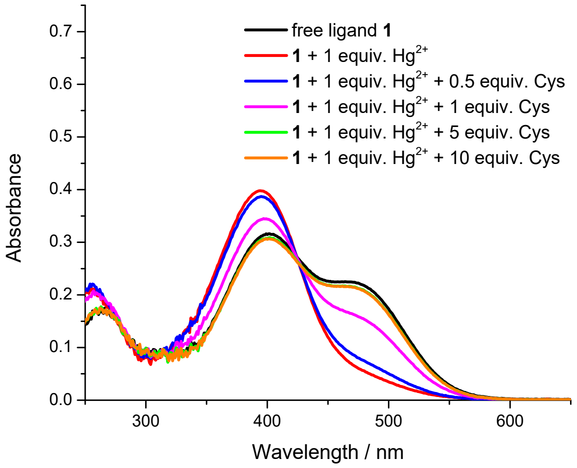

(**a**)

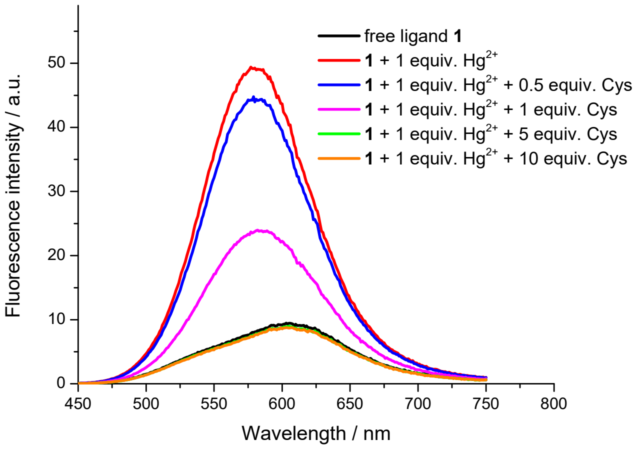

(**b**)

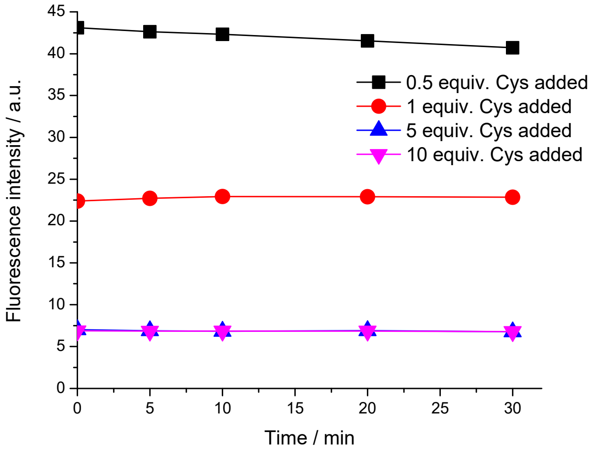

(**c**)

Figure S34. Changes in the absorption (**a**) and fluorescence (**b**) spectrum of compound **1** (10 μM) upon addition of 1 equiv. mercury (II) perchlorate followed by the addition of 0.5, 1, 5, and 10 equiv. cysteine (Cys) in water at pH 6.0 (acetate buffer, 0.01 M). Excitation wavelength was 380 nm. The interval between addition of aliqoutes of cysteine and collecting the spectra was 5 min. Panel (**c**) shows the time dependence of the fluorescence intensity at 570 nm for the solutions obtained after the addition of each aliqoute of cysteine.

13. Plot of Fluorescence Intensity Versus Increasing Concentrations of Hg^{2+} in Cells

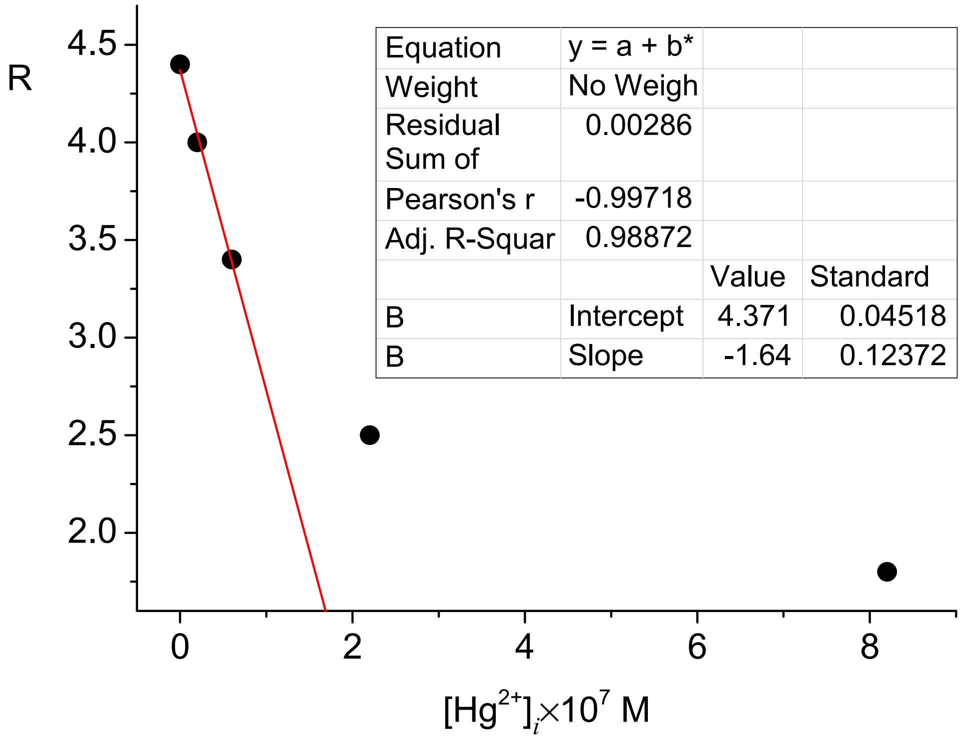

Figure S35. Plot of the ratio *R* of the fluorescence intensity in the yellow (500–600 nm) channel to that in the red channel (600–730 nm) for sensor **1** versus increasing intracellular concentrations of Hg^{2+}. Excitation wavelength is 405 nm. The insert shows the fitting parameters.

14. Detection of Hg^{2+} Concentration Changes in Cells

A549 cells were pre-incubated with 10 μM $Hg(ClO_4)_2$ for 20 min, washed twice with Hanks' solution, incubated with **1** (10 μM) for 30 min, washed twice with Hanks' solution, and placed in a fresh culture medium. Changes in intracellular concentration of Hg^{2+} were analyzed with sensor 1 in 0.5, 1, 2, and 3.5 h after removal of $Hg(ClO_4)_2$ from extracellular medium (Figure S35) on the basis of *I*(500–600 nm)/*I*(600–730 nm) ratio (*R*) using Equation (4).

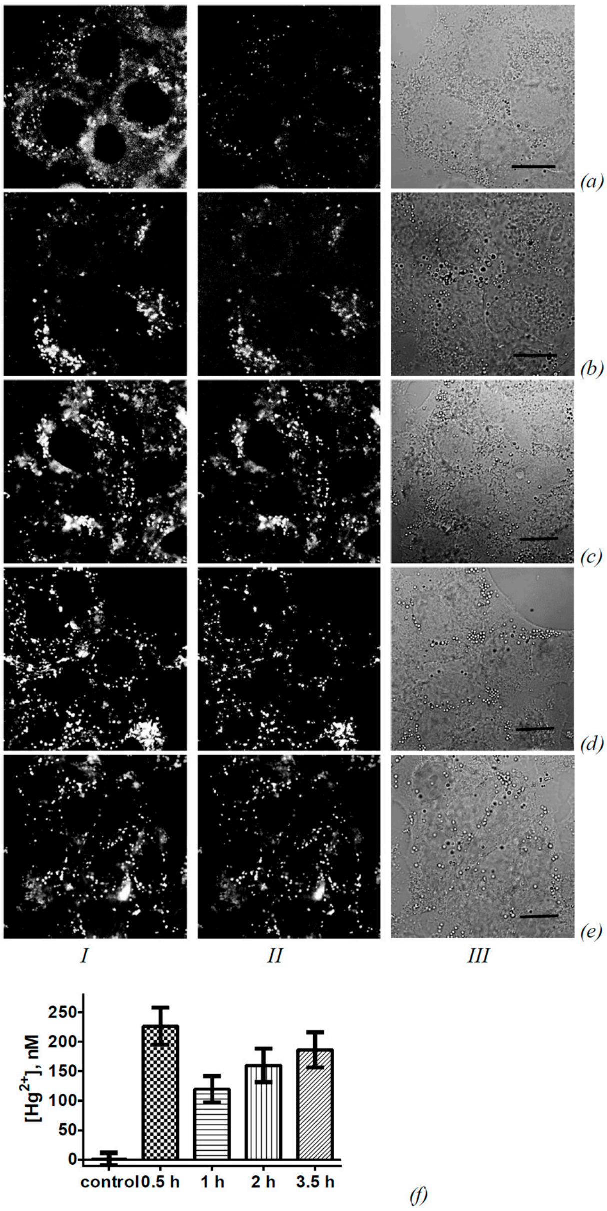

Figure S36. Analysis of changes in intracellular concentration of Hg^{2+} ions with sensor **1**. (**a**–**e**) Confocal fluorescence images of A549 cells stained with sensor **1** **(**10 μM, 30 min incubation**)** in the absence of Hg^{2+} (**a**) or after exposure to $Hg(ClO_4)_2$ (10 μM, 20 min) (**b**–**e**) are shown. (**b**–**e**) Images were measured in 0.5 (**b**), 1 (**c**), 2 (**d**), and 3.5 h (**e**) after removal of $Hg(ClO_4)_2$ from extracellular medium. Images of intracellular fluorescence were measured in the 500–600 nm (column I) and 600–730 nm (column II) spectral ranges at λ_{ex} = 405 nm. Transmitted light images of cells are shown in column III. Bar represents 10 μm. Details of cell treatment with **1** and $Hg(ClO_4)_2$ are described in the text. (**f**) Intracellular concentrations of Hg^{2+} were calculated using ratiometric approach in 0.5, 1, 2, and 3.5 h after removal of $Hg(ClO_4)_2$ from extracellular medium. Conditions of the experiment are described in the legend to panels (**a**–**e**). Control—cells in the absence of Hg^{2+}. The data are presented as the mean values ±SEM.

15. References

1. Lakowicz, J.R. *Principles of Fluorescence Spectroscopy*; Springer Science + Business Media, LLC: New York, NY, USA, 2006; ISBN 0387312781.
2. Stewart, J.J.P. Optimization of Parameters for Semiempirical Methods VI: More Modifications to the NDDO Approximations and Re-optimization of Parameters, *J. Mol. Model.* **2013**, *19*, 1–32, doi:10.1007/s00894-012-1667-x.
3. Efremenko, A.V.; Ignatova, A.A.; Grin, M.A.; Sivaev, I.B.; Mironov, A.F.; Bregadze, V.I.; Feofanov, A.V. Chlorin e6 fused with a cobalt-bis(dicarbollide) nanoparticle provides efficient boron delivery and photoinduced cytotoxicity in cancer cells. *Photochem. Photobiol. Sci.* **2014**, *13*, 92–102, doi:10.1039/c3pp50226k.
